# c-*Myc*-Induced Therapy Resistance in Leukemia: Mechanisms and Emerging Therapeutic Opportunities

**DOI:** 10.3390/medsci14020325

**Published:** 2026-06-16

**Authors:** Ali Rafat, Javad Arabpour, Haniye Yarahmadi, Hediye Khalkhali, Zeinab Mazloumi, Seyyede Sepide Ashraf Moosavi, Hossein Kalarestaghi, Khadijeh Dizaji Asl, Reza Nejati

**Affiliations:** 1 Anatomical Sciences Research Center, Institute for Basic Sciences, Kashan University of Medical Sciences, Kashan, Iran. 87159-87411; alirafat1370@gmail.com; 2 Department of Biophysics, Faculty of Biological Sciences, Tarbiat Modares University, Tehran, Iran. 14115-154; bio.javad.arabpour@gmail.com; 3 Department of Microbiology, Faculty of Basic Sciences, Tabriz Branch, Islamic Azad University, Tabriz, Iran. 51579-44511; haniye.yarahmadi@iau.ir (H.Y.); hediye.khalkhali3029@iau.ir (H.K.); 4 Department of Medical Applied Cell Sciences, Faculty of Advanced Medical Sciences, Tabriz University of Medical Sciences, Tabriz, Iran. 51579-44511; zb.mazloumi@gmail.com; 5 Student Research Committee, Kashan University of Medical Sciences, Kashan, Iran. 87159-87411; sepideh.amoosavi@gmail.com; 6 Research Laboratory for Embryology and Stem Cell, Department of Anatomical Sciences, School of Medicine, Ardabil University of Medical Sciences, Ardabil, Iran. 56189-58911; 7 Department of Histopathology and Anatomy, TaMS.C, Islamic Azad University, Tabriz, Iran. 51579-44511; 8 Department of Pathology, Fox Chase Cancer Center, Temple University Health System, Philadelphia, USA. PA 19111

**Keywords:** c-Myc, leukemia, oncogene, leukemogenesis, therapy resistance, targeted therapy

## Abstract

Dysregulation of the c-Myc oncogene is a pivotal event in leukemia pathogenesis and therapy resistance. This review synthesizes current evidence, illustrating that c-Myc drives leukemogenesis by enhancing proliferation, inhibiting apoptosis, and upregulating immune checkpoints like PD-L1. Its overexpression is linked to poor treatment outcomes across various leukemia subtypes. Directly targeting c-Myc remains challenging; however, indirect epigenetic modifiers (BET inhibitors), transcriptional disruption (CDK9 inhibitors), and combination therapies emerge as promising strategies to suppress its oncogenic activity and overcome resistance, paving the way for improved clinical management.

## 1. Introduction

Leukemia represents a heterogeneous group of hematologic malignancies characterized by uncontrolled proliferation and differentiation arrest of hematopoietic precursors in the bone marrow [1]. It is broadly categorized into four main types: acute lymphoblastic leukemia (ALL), acute myeloid leukemia (AML), chronic lymphocytic leukemia (CLL), and chronic myeloid leukemia (CML) [2]. According to the 2022 Global Reports, leukemia is the second most common hematological malignancy worldwide after non-Hodgkin lymphoma. In adults, AML is the most frequently diagnosed subtype, whereas in children, ALL is the most prevalent [3].

The pathogenesis of leukemia is fundamentally driven by the dysregulation of proto-oncogenes, normal cellular genes that regulate cell growth, division, and differentiation. When these genes become constitutively activated through chromosomal translocations, point mutations, or aberrant transcriptional activation, they drive uncontrolled proliferation, resistance to apoptosis, and clonal expansion [4]. Among these oncogenic drivers, the c-Myc transcription factor stands out as a central transcriptional hub that integrates multiple upstream signaling pathways to coordinate gene expression programs governing cell cycle progression, metabolism, and survival [5]. Its overexpression or dysregulation significantly contributes to leukemogenesis by promoting unrestrained cell proliferation and impaired cell death [6], and it is associated with adverse clinical outcomes and therapy resistance across all major leukemia subtypes [7].

The Myc oncogene family, comprising c-Myc (chromosome 8q24), N-Myc (chromosome 2p24), and L- Myc (chromosome 1p34), encodes basic helix-loop-helix leucine zipper (bHLH-Zip) transcription factors [8]. While these paralogs share similar structural motifs and functional roles, they differ significantly in their spatiotemporal expression patterns during development and cellular differentiation [9]. Among them, c-Myc is the most abundantly expressed and frequently dysregulated, with aberrations implicated in approximately 28% of all human cancers [9,10]. The c-Myc gene contains three exons, with protein-coding regions confined to exons 2 and 3; exon 1 remains untranslated [10]. Its oncogenic potential was first demonstrated through the identification of a Myc translocation to the immunoglobulin heavy chain (IGH) locus in Burkitt lymphoma, which results in its constitutive overexpression.

In leukemia, c-Myc drives malignant transformation through multiple convergent mechanisms. It promotes proliferation, inhibits terminal differentiation, reprograms cellular metabolism toward glycolysis and glutaminolysis, and confers resistance to apoptosis through interactions with BCL-2 family proteins [11,12]. Furthermore, c-Myc facilitates immune evasion by directly transactivating immune checkpoint molecules, notably programmed death-ligand 1 (PD-L1) and CD47, enabling leukemic cells to escape T-cell and macrophage-mediated immune surveillance [13,14]. c-Myc also enhances the expression of B-cell lymphoma 2 (Bcl-2), a well-known anti-apoptotic protein, thereby promoting the survival of malignant cells [15].

Beyond its cell-autonomous functions, c-Myc orchestrates a broader oncogenic network through extensive crosstalk with multiple signaling pathways. The PI3K/AKT/mTOR and RAS/RAF/MEK/ERK cascades converge on c-Myc to amplify its expression and transcriptional output [16]. Aberrant WNT/beta-catenin signaling promotes nuclear accumulation of beta-catenin, which directly activates Myc transcription [17]. Interleukin-6 (IL-6) upregulates c-Myc expression via JAK2/STAT3 signaling [18], while the NOTCH- c-Myc axis drives PD-L1 expression on leukemia cell surfaces, enabling immune evasion in CLL [19,20]. The clinical significance of c-Myc dysregulation is underscored by its consistent association with poor prognosis and resistance to both conventional chemotherapy and targeted agents [21]. Directly targeting c-Myc remains challenging due to its nuclear localization and lack of classical small-molecule binding pockets [22]; however, recent advances in indirect strategies, including BET bromodomain inhibitors, CDK9 inhibitors, and combination therapies, have renewed therapeutic optimism [23,24]. Therefore, this review aims to comprehensively synthesize current evidence on the role of c- Myc dysregulation in leukemia pathogenesis and therapy resistance, while evaluating emerging therapeutic strategies to counter its oncogenic activity and improve clinical outcomes.

## 2. c-Myc: Biology and Function

### 2.1. Structure and Regulation of c-Myc

c-Myc, as a major oncogene, encodes a transcription factor that regulates cell cycle, growth, and apoptosis. In addition to its nuclear function, c-Myc can also facilitate mitochondrial transport through tunneling nanotubes (TNTs) [25]. The hypoxia-inducible factor 1 (HIF1) transcript, along with c-Myc, pyruvate dehydrogenase kinase (PDHK), isocitrate dehydrogenase 2 (IDH2), and glutaminase 2 (GLS2), activates the glycolysis pathway [26]. Signaling through the PI3K/AKT/mTOR and RAS/RAF/MEK/ERK pathways regulates cellular metabolism by activating transcription factors such as c-Myc. c-Myc interacts with over 300 proteins and enhances RNA Pol II binding to gene promoters [27]. The Myc proto-oncogene family consists of three transcription factors encoded by genes located on chromosomes 8 (C-Myc), 2 (N-Myc), and 1 (L-Myc). Although these Myc paralogs share similar structural motifs and biological functions, their expression patterns vary spatially and temporally throughout the cell differentiation process [20]. c-Myc is a member of the basic helix-loop-helix leucine zipper (bHLH-Zip) family of transcription factors. It is localized in the cell nucleus, where it regulates growth, differentiation, metabolism, and cell death, and is involved in the pathogenesis of many human cancers. Among these family members, c-Myc is the most abundantly expressed, whereas N-Myc and L-Myc exhibit more limited expression profiles [28]. To become active, c-Myc forms a heterodimer with another bHLH protein called Max. This c-Myc/*Max* complex binds to E-box DNA sequences via its basic domain and recruits coactivator proteins such as histone acetyltransferases, which modify chromatin and promote gene transcription [29,30].

### 2.2. Normal Physiological Roles and Pathological Dysregulation of c-Myc

#### 2.2.1. c-Myc in Normal Physiology

Under physiological conditions, c-Myc functions as a tightly regulated transcription factor that heterodimerizes with MAX to orchestrate context-dependent gene expression programs [28]. Its expression is robust during embryonic development and in adult tissues with high proliferative capacity, such as the intestinal epithelium and hematopoietic progenitor compartments [31]. c-Myc promotes orderly cell cycle progression by driving the G0/G1 to S-phase transition, largely through transcriptional activation of cyclins, cyclin-dependent kinases, and repression of cell cycle inhibitors [32]. The essential nature of c-Myc in development is underscored by targeted deletion studies, where homozygous loss of c-Myc in mice results in embryonic lethality between days 9.5 and 10.5, accompanied by multi-organ developmental failure [33,34]. In addition to proliferation, c-Myc coordinates cellular metabolism by enhancing glycolytic flux and glutaminolysis, processes that provide biosynthetic precursors for growing cells [35]. c-Myc also regulates ribosome biogenesis and mitochondrial function, ensuring adequate energy production and macromolecular synthesis during cell growth [36]. Under normal conditions, c-Myc expression is self-limited through potent negative feedback mechanisms: its transcriptional activity is counterbalanced by pro-apoptotic signals mediated through the ARF/p53 and BIM/BAX pathways, while its protein stability is controlled by phosphorylation-dependent ubiquitination via the SCF-FBXW7 E3 ligase complex [37,38]. This intricate regulatory network ensures that c-Myc drives proliferation only when appropriate survival signals are present, preventing unscheduled expansion of progenitor populations [39].

#### 2.2.2. Dysregulation of c-Myc Functions in Cancer

In malignant contexts, the physiological checks on c-Myc activity are systematically dismantled through multiple convergent mechanisms [40]. Constitutive overexpression, gene amplification, or impaired protein degradation result in supraphysiological c-Myc levels that uncouple proliferation from survival signals [22]. Under these conditions, the pro-apoptotic arm of c-Myc signaling becomes a critical barrier to transformation, necessitating cooperative lesions that disable apoptotic checkpoints. Frequent co-occurring alterations include loss-of-function mutations in TP53, deletions of CDKN2A (encoding p14ARF and p16INK4a), activation of anti-apoptotic BCL2 family members, or deletion of pro-apoptotic mediators such as Caspase 8 [41,42]. These collaborative events allow cells to tolerate elevated Myc activity, enabling unrestrained proliferation while evading cell death [39]. Similarly, mutations or deletions in the FBXW7 ubiquitin ligase, which is responsible for Myc degradation, stabilize Myc protein and further amplify its oncogenic output [38]. Beyond cell-autonomous effects, dysregulated c-Myc reprograms the tumor microenvironment by upregulating immune checkpoint molecules including PD-L1 and CD47, promoting immune evasion and altering stromal interactions [23,24]. This pathological switch from a regulated transcriptional rheostat to a constitutively active oncogenic driver is central to the initiation, maintenance, and therapeutic recalcitrance of leukemia [9].

### 2.3. Mechanisms of c-Myc Activation in Leukemia

#### 2.3.1. Transcriptional Activation of c-Myc

The c-Myc oncogene is a critical regulator of cellular function and is implicated in the initiation and progression of various aggressive cancers, including leukemia. In AML, one of the key regulatory pathways of c-Myc expression is the exportin 1 (XPO1)/eukaryotic translation initiation factor 4E (eIF4E)/c-Myc axis, which plays a fundamental role in promoting cancer cell survival. A study by Chen et al. demonstrated that the combination of the XPO1 inhibitor Selinexor (KPT-330) and the hypomethylating agent Azacitidine synergistically reduced proliferation and induced apoptosis in AML cells. This outcome was mediated through the downregulation of XPO1, eIF4E, and most notably, c-Myc protein levels. Furthermore, c-Myc suppression enhanced the sensitivity of AML cells to this combination therapy. Clinical data indicate that elevated expression of XPO1 and eIF4E in AML patient samples correlates with poor prognosis, reinforcing the therapeutic significance of targeting this pathway. These findings highlight the critical role of the XPO1/eIF4E/c-Myc axis in AML progression and underscore the therapeutic potential of combining Selinexor and Azacitidine [43,44].

#### 2.3.2. Protein Stabilization and Post-Translational Regulation

Beyond the XPO1/eIF4E/c-Myc axis, multiple intracellular and extracellular mechanisms contribute to the aberrant activation of c-Myc in leukemia. A prominent mechanism involves the abnormal stabilization of the Myc protein due to impaired degradation pathways. Specifically, dysfunction in kinases such as Aurora kinase A (AURKA) and Polo-like kinase 1 (PLK1), which are normally involved in Myc degradation, results in Myc accumulation and sustained oncogenic activity. Preclinical studies targeting these kinases have shown promising efficacy in reducing Myc levels in malignant cells while sparing normal cells [40,45].

#### 2.3.3. Epigenetic Regulation and Synthetic Lethality

Emerging evidence also implicates epigenetic modifications in the sustained activation of c-Myc in leukemia. Enzymes such as histone deacetylases (HDACs), which influence chromatin remodeling [46], are involved in regulating Myc expression and the transcription of Myc-responsive genes. Pharmacological inhibition of HDACs particularly when combined with other therapies can suppress Myc-driven oncogenic signaling and restore antitumor immunity by activating interferon pathways and enhancing immune cell infiltration into tumors [47]. Another notable mechanism is synthetic lethality; wherein Myc-dependent leukemia cells exhibit a heightened reliance on specific survival pathways. Inhibiting both Myc and these auxiliary pathways leads to a collapse in cellular viability [48]. Additionally, Myc contributes to immune evasion by modulating the expression of multiple immune checkpoint molecules, including PD-L1 and CD47. It has been shown that Myc can directly bind to the promoter region of the Cd274 gene (encoding PD-L1), thereby upregulating its transcription a mechanism validated in murine leukemia models [49].

### 2.4. Crosstalk with Other Signaling Pathways

**Figure 1:**
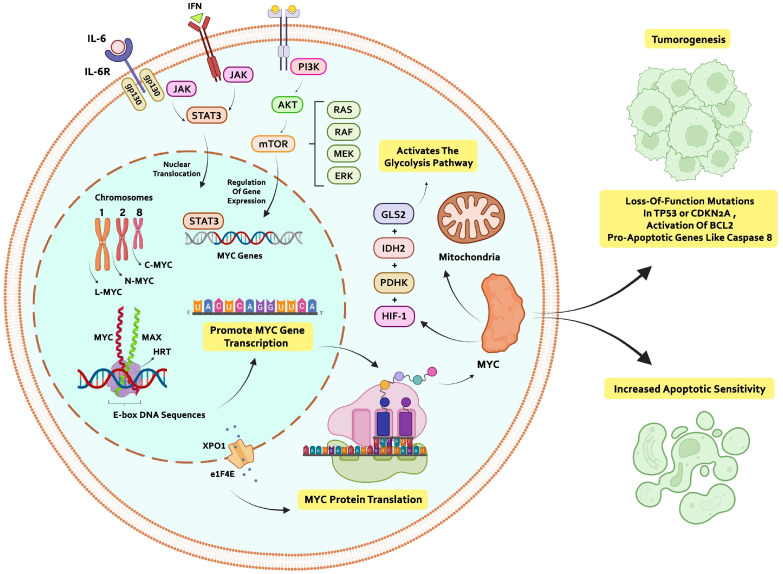
Integrated signaling networks regulating c-Myc transcription, translation, metabolic reprogramming, and apoptotic responses in cancer cells. The main upstream routes and intracellular regulatory nodes that regulate c-Myc activation in both healthy and malignant settings are depicted in this schematic. Growth factor signaling triggers the PI3K/AKT/mTOR and RAS/RAF/MEK/ERK cascades, which together increase Myc gene transcription, whereas cytokine-mediated stimulation (such as IL-6/IL-6R and IFN) activates the JAK/STAT3 axis. Encoded on chromosomes 8, 2, and 1, respectively, the Myc family (C-Myc, N-Myc, and L-Myc) generates bHLH-Zip transcription factors that heterodimerize with MAX and bind E-box motifs to promote expression of genes controlling metabolism, proliferation, and survival. c-Myc protein levels in the cytoplasm are further regulated via eIF4E-mediated cap-dependent translation and XPO1-dependent nuclear export. In order to sustain anabolic growth demands, c-Myc rewires cellular metabolism downstream of oncogenic signaling by triggering glycolytic and mitochondrial pathways via regulators such PDHK, IDH2, GLS2, and HIF-1. Oncogenic settings frequently contain loss-of-function mutations in TP53 or CDKN2A, activation of BCL2, or deletion of pro-apoptotic mediators like Caspase-8, allowing cells to evade checkpoints and drive carcinogenesis, even if physiological c-Myc expression boosts apoptotic sensitivity. These interrelated pathways show how immunological evasion, resistance to apoptosis, metabolic adaptability, and malignant transformation are all facilitated by dysregulated c-Myc. Created in BioRender. Keyhani, A. https://BioRender.com/aeesa7j (accessed on 16 May 2026). Agreement number: XF29Q11M9Y.

The expression and translation of c-Myc are modulated by various intracellular signaling pathways. Under normal physiological conditions, APC and GSK-3β function to degrade β-catenin; however, in malignant states where the WNT signaling pathway is dysregulated, β-catenin accumulates in the nucleus and promotes c-Myc transcription [50]. Interleukin-6 (IL-6) also contributes to the upregulation of c-Myc expression by activating the JAK2/STAT3 signaling cascade, which simultaneously increases the expression of metabolic enzymes such as GLUT1 and LDHA [51]. Recent findings have shown that, under cellular stress, the dimeric form of PKM2 promotes internal ribosome entry site (IRES)-mediated translation of c-Myc, a mechanism that plays a key role in glutamine metabolism regulation [52]. RNA-binding proteins such as DDX6 further enhance glycolysis and tumor proliferation by stimulating c-Myc translation. In contrast, miR-124 suppresses glycolysis by altering pyruvate kinase conformation and downregulating c-Myc production [53]. Additionally, CD36 has been shown to inhibit β-catenin signaling through suppression of glypican-4 (GPC4), thereby reducing c-Myc expression [54]. In leukemia, direct interactions between leukemic and stromal cells activate the NOTCH–C-Myc axis, which in turn induces PD-L1 expression on cancer cell surfaces, enabling CLL cells to evade immune surveillance by inhibiting T cell activity [55,56]. c-Myc also contributes to immune evasion by repressing microRNAs such as miR-26a and miR-101, leading to increased expression of EZH2. EZH2 not only mediates histone methylation but also enhances the transcription of c-Myc and PD-L1, creating a positive feedback loop in cancer progression [57,58]. Inhibiting c-Myc directly using small molecules like 10058-F4 has been shown to reduce both c-Myc and PD-L1 levels, thereby enhancing immune response against leukemia cells [59]. Moreover, the MAPK and PI3K/AKT/mTOR pathways upregulate c-Myc expression, subsequently activating immunosuppressive mechanisms, including PD-L1 and CD47 upregulation, which allow cancer cells to escape immune destruction [24,60] (Figure 1).

Oncogenic cooperation between KRAS and c-Myc accelerates T-cell leukemia (TLL). Notably, KRAS mutations amplify the leukemogenic potential of c-Myc, even in the presence of weak Notch1 signaling, underscoring a synergistic relationship between these oncogenes [61].

Additionally, cisplatin resistance in leukemia has been linked to increased levels of cyclin E and c-Myc, driven by p21 suppression, which promotes cell cycle progression and survival [62].

In the context of elevated c-Myc expression, the presence of weak Notch1 alleles has been shown to further accelerate leukemogenesis, highlighting a synergistic relationship between these two oncogenic pathways [63]. Conversely, reduced expression of c-Myc has also been associated with the development of drug resistance, as low c-Myc levels render leukemic cells less responsive to apoptosis-inducing agents, including tumor necrosis factor-alpha (TNF-α) and related death signaling pathways [64]. The simultaneous involvement of multiple signaling networks such as JAK2, KRAS, c-Myc, SAT-2, and p53 has been demonstrated in models of chemically induced leukemia using 7,12-dimethylbenz[a]anthracene (DMBA). This supports the concept of functional interdependence among these oncogenic and tumor suppressor pathways in leukemogenesis [61]. Furthermore, hypoxic conditions stimulate the expression of c-Myc through activation of hypoxia-inducible factor 2-alpha (HIF-2α). This hypoxia-driven c-Myc upregulation enhances the proliferative capacity of cancer cells, thereby promoting tumor progression in low-oxygen environments [65].

## 3. c-Myc in Leukemia Pathogenesis

### 3.1. Role in Leukemogenesis

The studies show that c-Myc is a key transcription factor that plays an important role in leukemogenesis. Activation of c-Myc in leukemia can occur through various pathways, such as gene mutations, epigenetic changes, and environmental signals. Increased expression of c-Myc leads to changes in cellular properties, such as rapid proliferation, resistance to apoptosis, and cell migration. Targeting c-Myc or its associated signaling pathways could be explored as a therapeutic strategy in leukemogenesis [66].

In patients with AML, the expression level of c-Myc is significantly increased compared to healthy individuals. This increase is accompanied by increased expression of FLT-3 and STAT3 genes and decreased expression of p27. Evidence suggests that FLT-3 may inhibit p27 through the c-Myc pathway, thereby enhancing the proliferation and survival of AML cells. In addition, a long non-coding RNA (lncRNA) called HOX transcript antisense RNA (HOTAIR) is also involved in inducing proliferation of leukemic cells, which is likely exerted through increased expression of STAT3 [67].

In a mouse model of CLL, c-Myc expression is elevated in PKCα-KR cells compared to controls. Phosphorylation of c-Myc at serine-62 (S62) is also increased, indicating greater stability of the protein. This upregulation of c-Myc and its phosphorylation is accompanied by activation of the AKT/mTOR and BCR-signaling pathways, as well as increased phosphorylation of proteins such as BTK at positions Y551 and Y223, indicating continued BCR signaling activity [68].

In acute promyelocytic leukemia cells, epigenetic reprogramming results in widespread repression of c-Myc-dependent pathways and a decrease in the expression of this oncogene at the mRNA and protein levels. These changes indicate that c-Myc plays a central role in maintaining the leukemic phenotype, and its downregulation limits uncontrolled proliferation and inhibits the leukemogenesis process. Furthermore, c-Myc repression is accompanied by silencing of its target genes and activation of viral-like and interferon-like responses, which may contribute to the potentiation of the antileukemic effect and inhibition of disease progression [69]. On the other hand, in ALL, disruption of the IKZF1 gene, which encodes the transcription factor IKAROS, is associated with increased expression of c-Myc. Normally, IKAROS inhibits c-Myc expression by binding to the promoter region, but deletions or loss-of-function mutations in IKZF1 weaken this inhibition and cause excessive activation of c-Myc. In patients with ALL, such alterations have been associated with high levels of c-Myc and reduced expression of Myc BP2. This condition exacerbates the proliferation of B-cell progenitor cells and blocks their differentiation, thus making c-Myc a key driver in the leukemogenesis process [70].

In B-ALL samples, the expression of HBO1, a histone acetyltransferase enzyme, is significantly increased, and this increase is associated with worse patient survival. HBO1 increases the expression of CTNNB1 through the acetylation of histones H3K14, H4K8, and H4K12, thereby activating the Wnt/β-catenin signaling pathway. Activation of this pathway is associated with increased expression of c-Myc and its downstream genes such as Cyclin D1 and MMP7, leading to enhanced cell proliferation, inhibition of apoptosis, and continuation of the leukemogenesis process. Evidence from animal models has also shown that silencing HBO1 inhibits tumor growth and reduces the levels of c-Myc and its targets [71]. In patients with AML with FLT3-ITD mutations, c-Myc gene expression levels are significantly higher than in groups without this mutation, suggesting a possible role for c-Myc in the pathogenesis of FLT3-ITD-associated AML. While increased N-Myc expression is observed in these patients, this increase is poorly correlated with the allelic level of the FLT3-ITD mutation, which may indicate FLT3-independent pathways for N-Myc activation [72].

### 3.2. Influence on Cell Proliferation, Apoptosis, and Metabolism

Myc is a transcription factor that controls immune cell formation and proliferation. Myc regulates cell metabolism by regulating a metalloproteinase transcriptome that occurs before cell cycle entry and after antigenic stimulation [35]. Overexpression of Myc in leukemia cells leads to cell proliferation, resulting in 42.1% more S phases in the cells than in the control group. Through metabolic programming, Myc stimulates the division of cancer cells by directly activating genes related to glycolysis, nucleotide biosynthesis, and ribosome biogenesis. By producing pro-apoptotic proteins like PUMA and BIM and by downregulating the expression of the anti-apoptotic BCL-2, Myc makes cells more susceptible to apoptosis. A shock, like growth factor deprivation or DNA damage, is necessary for Myc to induce apoptosis in order to block survival signaling [36]. It is believed that c-Myc and c-Myb regulate cell proliferation in the myeloid lineage via distinct routes; nonetheless, inhibiting c-Myb can diminish c-Myc and, as a result, restrict myeloid cell proliferation. Studies have revealed that exposing of myeloid HL-60 cells to anti c-Myc oligomers reduces cell growth. As a result, it is possible that lowering the level of c-Myc protein alone inhibits HL-60 cell proliferation [73]. One of the primary transcriptional regulators, c-Myc, is dysregulated in over 70% of malignancies and speeds up the development of tumors by promoting cell division, immune evasion, and metabolic survival. Experimental evidence demonstrates that Myc inactivation in ALL cells induces apoptotic cell death and suppresses cellular proliferation. Notably, PAK4 phosphorylates S67 to maintain the level of Myc protein, while FBXW7 ubiquitination boosts AML cell survival [74]. Immunoblotting experiments show that when PAK4 is inhibited, Mcl-1, a survival factor, rises. In actuality, ALL cells are resistant to apoptosis due to the stability of Myc with elevated Mcl-1. Additionally, leukaemia stem cells can survive but not differentiate when Smad2/3 is phosphorylated by inhibiting p21 and c-Myc expression. According to Gene Set Enrichment Analysis (GSAE) investigations, AML cells exhibit an overactive metabolic state, as evidenced by the upregulation of ribosome and metabolism-related pathways. By interacting with the BAX/BCL2 pathways, c-Myc can also increase resistance to apoptosis and enhance survival [75,76]. We discovered that glucose deprivation causes growth inhibition, cell cycle arrest, and widespread apoptosis in erythroleukemic cells, as well as downregulation of c-Myc and HK2, which are responsible for glucose metabolism [77].

It should be noted that c-Myc is one of the molecules involved in BCR-ABL signaling and is great importance for the survival and regulation of malignant cells. Inhibition of c-Myc in the G1 phase by P1 and P2 can promote cell cycle progression in K562. Suppression of c-Myc and its genes, including PP2A, CIP2A, and hTERT, can reduce metabolic activity in K562 cells. Research suggests that c-Myc can alter the balance between pro- and anti-apoptotic members of the BCL-2 family [78].

### 3.3. c-Myc Expression Patterns in Different Leukemia Subtypes (e.g., AML, CML, ALL)

Gene expression profiling has enabled precise characterization of molecular heterogeneity in AML. Distinct expression patterns correspond to cytogenetic and molecular subtypes, including inv (16), t (8;21), t (15;17), and CEBPA mutations and can identify novel subgroups even among cases lacking classical abnormalities. Importantly, pediatric derived expression signatures can classify adult AML cases with the same lesions, highlighting the robustness of this approach and its relevance for refined molecular diagnosis and risk stratification [79].

In different leukemia subtypes, c-Myc acts as a key transcription factor regulating cell growth and proliferation, with distinct expression patterns observed across AML, ALL, and CML. In AML, elevated c-Myc expression is associated with activation of survival and proliferation pathways and with resistance to therapy, often in combination with genetic alterations such as FLT3-ITD or other growth-regulatory gene changes. In ALL, c-Myc is frequently activated through chromosomal abnormalities, including Myc translocations or copy number gains, correlating with aggressive clinical behavior and poor prognosis. In CML, c-Myc is regulated indirectly via BCR-ABL1 activated signaling pathways, contributing to disease progression and genomic instability [80]. In the Egyptian AML cohort, overall c-Myc mRNA expression did not significantly differ between AML patients and healthy controls. However, when FAB subtypes were compared, patients with M4/M5, exhibited lower c-Myc expression compared to those with M0–M2 subtypes and controls [81].

In AML, c-Myc expression has been shown to vary across different patient subgroups. Bioinformatic analyses of TCGA-LAML and GSE71014 datasets revealed differential expression of c-Myc and Cyclin D1, suggesting their involvement in leukemia cell proliferation and survival. Experimental results further indicated that suppression of CD4 led to decreased c-Myc expression, highlighting the critical role of c-Myc in regulating apoptosis and ferroptosis pathways in AML cells [82].

Long non-coding RNAs (lncRNAs) have emerged as critical regulators in leukemia, influencing disease progression, therapeutic response, and clinical outcome. Distinct lncRNA expression profiles are observed across AML, ALL, CML, and CLL, reflecting subtype-specific molecular mechanisms. In AML and ALL, aberrant lncRNA expression is associated with altered cell proliferation, apoptosis, and chemotherapy resistance, while in CML, lncRNAs modulate BCR-ABL1-driven signaling pathways, contributing to disease progression. These molecules not only serve as potential diagnostic and prognostic biomarkers but also represent promising therapeutic targets [83]. Compared to AML, CML, and CLL, UHRF1 is significantly overexpressed in ALL at both the protein and mRNA levels. Increased expression of c-Myc, CDK4, and CDK6 is correlated with enhanced UHRF1 expression. In B-ALL and T-ALL, functional knockdown of UHRF1 results in decreased cell survival, along with decreased levels of c-Myc protein, CDK4/CDK6, and Rb phosphorylation, indicating that UHRF1 controls ALL growth through the c-Myc-CDK4/6-pRb pathway [84]. In adult T-cell leukemia/lymphoma (ATL), treatment of HTLV-1 infected T-cells with the natural compound Conferone resulted in a marked reduction of cell viability. This effect was accompanied by the downregulation of c-Myc, CDK6, c-FLIPL, and NF-κB (RelA), suggesting that suppression of c-Myc expression contributes to the antileukemic activity of Conferone in ATL cells [85].

Furthermore, the prognostic value of Myc expression has been extended to other hematological malignancies. In diffuse large B-cell lymphoma, Myc overexpression detected by immunohistochemistry has been correlated with poor clinical outcomes, highlighting the broader relevance of Myc as a prognostic biomarker across B-cell neoplasms [86]. The key mechanisms and functional consequences of c-MYC dysregulation in different leukemia subtypes are summarized in Table 1.

### 3.4. c-Myc Impact Leukemia Stem Cells and Disease Progression

The WNT/β-catenin pathway transcriptionally activates multiple target genes, notably c-Myc. Hydroquinone (HQ) promotes Wnt/β-catenin signaling through SUV39H1-mediated accumulation of H3K9me3, resulting in upregulated expression of c-Myc, WNT2B, β-catenin, and CCND1, alongside downregulation of WNT5A. All of these alterations promote cell development while reducing apoptosis [89]. By inhibiting BATF3 in CML cells, c-Myc, which is crucial for the development and multiplication of cancer cells as well as for the entry of cells into the S and G2/M phases, has been demonstrated to induce apoptosis and cell cycle arrest. These investigations employed siRNA to decrease the transcription factor BATF3 expression in K562 CML cells. These findings may represent novel treatment strategies for CML [90].

Cyclin-dependent kinase 8 (CDK8), one of the transcriptional CDK family’s members is CDK8. The lack of tumor growth ability stems from genetic suppression of CDK8 by shRNA or CRASPR, which is linked to the artificial expression of c-Myc [91].

Overexpression of c-Myc causes fibroblasts to become functional macrophages, and those fibroblasts that experience excessive c-Myc expression rapidly produce myeloid (MCC) CD45+ cells. Studies show that c-Myc causes the differentiation of cells into macrophages through the increase in MafB. These macrophages induced by c-Myc inhibit leukemia [92].

The functional interaction between c-Myc and the hypoxia-inducible factor (HIF) pathway represents a paradigm of context-dependent crosstalk with profound implications for leukemia biology. Under normoxic conditions, c-Myc functions as a master regulator of proliferation and metabolism, driving cell cycle progression and anabolic pathways. Under moderate hypoxia, HIF-1alpha antagonizes c-Myc activity through multiple mechanisms: it transcriptionally induces the Myc antagonist MXI1, promotes proteasomal degradation of c-Myc, and directly competes for binding to shared transcriptional cofactors such as SP1[65]. This HIF-1alpha-mediated suppression constrains Myc-driven proliferation and biosynthetic metabolism, adapting cells to reduced oxygen availability. However, under chronic or severe hypoxia, a condition prevalent in the leukemic bone marrow niche, a functional switch occurs. HIF-2alpha, which is stabilized under prolonged hypoxic stress, physically interacts with c-Myc/MAX complexes at target gene promoters, enhancing rather than repressing transcriptional activity [65,93]. This HIF-2alpha/c-Myc cooperative axis upregulates glycolytic enzymes such as hexokinase 2 (HK2) and lactate dehydrogenase A (LDHA), glucose transporters such as GLUT1, and stemness factors to promote metabolic adaptation and maintenance of leukemia stem cells (LSCs) [26,52]. In LSCs residing within the endosteal niche, where oxygen tension is markedly low, this HIF-2alpha-driven enhancement of c-Myc function is particularly critical: it simultaneously drives quiescence exit, self-renewal capacity, and apoptosis resistance through upregulation of BCL2 and MCL-1 [93]. Thus, c-Myc function shifts from being suppressed by HIF-1alpha under moderate hypoxia to being potentiated by HIF-2alpha under severe hypoxia. This functional divergence has important therapeutic implications: targeting HIF-2alpha selectively, or disrupting the HIF-2alpha/c-Myc interaction, may preferentially eliminate LSCs while sparing normal hematopoietic stem cells, which rely predominantly on HIF-1alpha-mediated adaptive responses under hypoxia [65,93]. It has been determined that c-Myc mRNA is bound by RNA-binding proteins (RBPs), which regulate its translation and stability. For example, IGF2BP proteins promote carcinogenesis and stabilize Myc transcripts in leukemic cells. Since Myc’s role in leukemia may be inhibited by disrupting RBP-mRNA and Myc interactions, targeting Myc indirectly through RBPs is also a promising strategy [94].

In the clinical setting, the persistence of CD34+CD38- leukemic stem cells following chemotherapy has been identified as a marker of poor therapeutic response in AML patients, underscoring the translational relevance of LSC-targeted strategies [95].

### 3.5. Differential Roles of Myc Paralogs in Leukemia and Leukemia Stem Cells

While c-Myc is the predominant paralog implicated in leukemia pathogenesis, emerging evidence reveals distinct and complementary roles for N-Myc and L-Myc in specific disease contexts. c-Myc is ubiquitously expressed across hematopoietic lineages and serves as a universal driver of proliferation and metabolic reprogramming in virtually all leukemia subtypes [9]. Its central role in leukemia stem cell (LSC) maintenance is well-established: conditional deletion of c-Myc in murine AML models eradicates LSCs and abrogates leukemia-initiating capacity, highlighting a non-redundant function that cannot be compensated by other Myc paralogs [73].

N-Myc, traditionally associated with neuroendocrine and pediatric solid tumors, is increasingly recognized as an important contributor in specific AML subsets. In FLT3-ITD-mutated AML, N-Myc expression is significantly elevated, though its expression correlates poorly with the FLT3-ITD allelic ratio, suggesting FLT3-independent mechanisms of activation [72]. Notably, in KMT2A (MLL)-rearranged leukemias, N-Myc is a direct transcriptional target of the KMT2A fusion complex and contributes to the aggressive clinical behavior of this subtype [91]. In these contexts, N-Myc drives transcriptional programs that partially overlap with c-Myc but also uniquely regulate genes involved in oxidative phosphorylation and mitochondrial biogenesis [35].

L-Myc remains the least characterized paralog in leukemia. Limited studies have reported L-Myc expression in a subset of B-ALL cases, where it may function as a weaker transcriptional activator compared to c-Myc [9]. Unlike c-Myc, L-Myc lacks a conserved phosphorylation site critical for FBXW7-mediated degradation, rendering it resistant to ubiquitin-dependent proteolysis and potentially contributing to sustained signaling under conditions where c-Myc would be degraded [38].

In LSCs, the paralog-specific contributions appear hierarchical: c-Myc is the dominant regulator of self-renewal and proliferation, N-Myc supports metabolic adaptation under specific oncogenic drivers, and L-Myc may serve as a backup mechanism when c-Myc is therapeutically suppressed [93]. This functional redundancy has important implications for therapeutic targeting; complete Myc inhibition may require pan-Myc strategies to prevent paralog-mediated resistance, particularly in LSC populations where selective pressure is highest [66].

## 4. c-Myc’s Interaction with Epigenetic Modifiers

According to a study by Zhang et al., the deubiquitinating enzyme USP1 can bind directly with c-Myc and decrease its ubiquitination, which increases the stability of the c-Myc protein and improves the c-Myc pathway’s functionality. Since the inactive mutant form of USP1 cannot stabilize c-Myc, this interaction depends on USP1’s catalytic activity. Cycloheximide experiments further shown that USP1 increases the half-life of c-Myc, whereas the USP1 mutant is unable to replicate this effect. Additionally, USP1 knockdown led to lower levels of c-Myc and less tumor formation in animal models because it raised the level of c-Myc ubiquitination in experimental cells [96]. Many malignancies have overexpression of c-Myc, a crucial transcription factor that controls metabolism, proliferation, and survival. Research has demonstrated that NKLAM expression dramatically lowers c-Myc stability and abundance. Although c-Myc is not ubiquitinated, this decrease is proteasome-dependent. The 20S proteasome can directly destroy c-Myc due to its intrinsically disordered structure; this action is aided by small-molecule activators or regulatory subunits like PA28γ. Therefore, c-Myc instability is a result of NKLAM-mediated proteasomal activity modulation, which is an indirect epigenetic-like mechanism that regulates proliferation and makes cells more susceptible to death [97]. Single nucleotide polymorphisms, insertions, deletions, and copy number variations are examples of genomic changes that can impair normal gene activity and change how proteins are expressed. Deregulated cell proliferation and genomic instability are two outcomes of these alterations that are essential to the development of cancer. Malignant transformation may be promoted in this situation by mutations that either activate oncogenes like c-Myc or deactivate tumor suppressor genes. By shedding light on these changes, advances in genomic research have made it possible to pinpoint the precise molecular processes that underlie carcinogenic pathways. Understanding how c-Myc and associated variables contribute to carcinogenesis has also helped to advance precision medicine strategies that target the genetic and epigenetic regulators of these oncogenes [98]. Sox6 and Fnip1 activity are closely related to the control of c-Myc expression. Under normal circumstances, Fnip1 interacts with phosphorylated STAT3 to prevent it from inducing c-Myc production, whereas Sox6 attaches to an upstream area of the c-Myc gene to suppress its transcription. Elevated c-Myc levels result from the disruption of this repressive regulation caused by deletion of either Sox6 or Fnip1. By increasing the expression of GLUT1 and GLUT3, this upregulation promotes glucose absorption and glycolytic metabolism. Furthermore, by inhibiting regulators like MyoD and MyoG, high c-Myc activity leads to poor myoblast development [99].

### Emerging Biomarkers Linked to c-Myc Activity

According to recent research, the CNOT2 protein can influence the control of c-Myc expression because it is a part of the CCR4-NOT complex. In particular, ribosomal proteins including RPL5 and RPL11, which can suppress MDM2 function and indirectly raise cell sensitivity to apoptosis by stabilizing p53, are elevated in response to CNOT2 knockdown. Furthermore, these ribosomal proteins, RPL5 and RPL11, have the ability to attach to the RISC complex and degrade c-Myc messenger RNA, which in turn lowers c-Myc activity. Inhibiting CNOT2 increases the repressive effect on c-Myc. for instance, c-Myc expression is greatly decreased in liver cells HepG2 and Huh7 when CNOT2 is decreased [100]. The expression of c-Myc and its phosphorylated version may be new biomarkers for tracking this factor’s function, according to recent data. These two markers can accurately depict the actual level of c-Myc activity and, because they are objective and quantitative, enable a more accurate evaluation of the molecular alterations linked to this protein [101]. Also through the EBF3/RPL11/c-Myc axis, SNORA47 is essential for preserving the characteristics of cancer stem cells and controlling chemotherapy sensitivity. By causing RPL11 to move from the nucleolus to the nucleoplasm, it interacts with EBF3, which keeps more RPL11 in the nucleolus and lessens its ability to inhibit c-Myc. Because c-Myc levels are higher in these circumstances, it is more active in encouraging drug resistance and self-renewal. As a result, SNORA47 is discovered to be a new biomarker associated with c-Myc activity, offering more information on the regulatory pathways that link non-coding RNAs with oncogenic signaling [102].

## 5. Recent Research Advances

### 5.1. Novel Insights into c-Myc Regulation and Stability

In the analysis of c-Myc two consecutive phosphorylation, serine 62 (S62) and Tp 58, are involved. Following cell growth stimulation, ERK or CDKs phosphorylate Myc at ps62, which sets off further phosphorylation by GSK-3β. Protein phosphatase 2A can eliminate the phosphorylation at ps62, phosphorylating Myc, which is subsequently ubiquitinated by FBXW7 and broken down by the 26s proteasome. Additionally, abnormal PI3K/AKT activation causes GSK3β to become phosphorylated, which in turn causes c-Myc to become inactive. The results showed that increase of Myc occurs when the tumor suppressor gene PTEN is inactivated via PI3K/AKT [37].

E3 ligase, the primary ligase in charge of breaking down the Myc protein, is found in mutations in a considerable percentage of human malignancies, including T-ALL, and is encoded by the FBXW7 gene. Consequently, a study found that GFP- Myc and FBXW7 knock-in mouse models were significantly dependent on each other. These results show that FBXW7 is essential for maintaining Myc. Additionally, research has demonstrated an aberrant process involving Myc modification and proteasomal degradation, whereby Aurora kinase B (AURKB) binds to Myc and phosphorylates it directly at serine 67 (S67). By blocking the connection between Myc and GSK3β, this phosphorylation inhibits the phosphorylation of TP58. The carcinogenic characteristics of Myc are enhanced when stabilized Myc and T-cell acute lymphoblastic leukemia factor 1 (TAL1) activate AURKB expression [38]. IGH remodeling can also be improved by c-Myc. In addition to its well-established carcinogenic effects on cell cycle progression, c-Myc also seems to have other roles, including causing genetic recombination and DNA damage. Future research may ascertain how high amounts of c-Myc impact coronalin and modify transcriptional pathways in CLL, as all of this evidence points to the role that c-Myc plays in promoting carcinogenesis [103]. By influencing immune cell polarization through epigenetic variables and mechanisms, lncRNAs offer a new perspective on the control and stability of c-Myc. In order to attract EP300 to the nucleus, for instance, lncRNA PACERR interacts with KLF12; this increases histone acetylation and activates the KLF12/c-Myc/p-AKT pathway, which aids in M2 macrophage development [104]. The polarization of tumor-associated macrophages is increased when PACERR binds to CTCF and attracts p300 to the promoter regions of PACERR and PTGS2. Through lncRNA activation, c-Myc suppresses p53, while MILIP binds to TRIML2 and inhibits it. These occurrences cause p53 to degrade and become more polyubiquitinated [105].

### 5.2. Advances in Targeting c-Myc Indirectly

Indirect methods for inhibiting c-Myc have attracted considerable attention, as they circumvent the structural challenges of directly targeting this transcription factor. Among these, bromodomain and extra-terminal (BET) inhibitors represent the most extensively studied class, functioning by displacing BRD4 from hyperacetylated chromatin at the Myc locus, thereby suppressing Myc transcription [106]. While first-generation BET inhibitors such as JQ1 demonstrated robust preclinical efficacy, clinical translation has been limited by dose-limiting toxicities and the development of resistance [107]. Recent efforts have therefore shifted toward next-generation BET inhibitors that target non-bromodomain regions, including the ET domain and intrinsically disordered regions (IDRs), enabling more selective modulation of BET-dependent transcription [108]. CDK9 inhibitors offer a complementary approach by disrupting transcriptional elongation of Myc and its target genes. The CDK9 inhibitor KB-0742 has entered early-phase clinical trials, showing promising activity in reducing Myc expression, though off-target effects remain a concern due to the broad role of CDK9 in global transcription [107]. Voruciclib, another CDK9 inhibitor, has been shown to reduce c-Myc and MCL-1 levels in AML cell lines and patient samples, and is currently being evaluated in a phase I clinical trial for relapsed/refractory AML and B-cell malignancies [109]. An alternative indirect strategy involves stabilizing the G-quadruplex structure within the c-Myc promoter, which prevents transcription factor binding and reduces gene expression [110]. Similarly, disrupting the c-Myc/MAX interaction or preventing their binding to E-box sequences has been proposed as a therapeutic strategy, though small molecules targeting this interface, such as 10058-F4, have shown limited clinical utility due to poor pharmacokinetic properties [111,112]. The clinical evaluation of these indirect approaches is discussed in detail in Section 6.

## 6. Therapeutic Strategies Targeting c-Myc

### 6.1. Challenges in Directly Targeting c-Myc

Direct targeting of c-Myc has always been contentious. Recent studies have demonstrated that small molecule stabilisers of G-quadruplex structures are effective against c-Myc. A phase 1b study of APTO-253 in patients with relapsed/refractory acute myeloid leukemia (AML) or high-risk myelodysplastic syndromes (MDS). According to previous studies, BRD4 degradation induced by DBET1 leads to reduced Myc transcription. The c-Myc protein lacks a particular binding site for other molecules, rendering its location in the nucleus inaccessible to monoclonal. Small compounds like F4-10058 have been discovered to block the dimerization of c-Myc and MAX protein, making AML cells more sensitive to cytotoxic treatments. However, there are still structural barriers to targeting c-Myc [106,113]. c-Myc is unmodifiable due to its lack of small molecule binding sites and placement in the nucleus. To suppress c-Myc, investigations are being undertaken on the JAK/STAT, PIK3/AKT/mTOR, and CDK09 pathways [112]. In most situations, ligand binding to the G-quadruplex is accomplished via multi-step synthetic approaches with low efficiency. Furthermore, the TMPyP4 molecule cannot differentiate between distinct G-quadruplex structures and has limited attraction for them. Cancer cells develop resistance, which causes instability in the G-quadruplex and reduces therapeutic efficacy and to obtain optimum therapeutic efficacy, ligands must precisely bind to the G-quadruplex [38,114].

### 6.2. Indirect Strategies

#### 6.2.1. Small Molecule Inhibitors

Significant advancements have been achieved in the creation of small molecule inhibitors that target c-Myc in malignancies in recent years. These inhibitors decrease the expression of c-Myc target genes, which stops tumor growth and improves the response to immunotherapy. As an illustration, the new immunoconjugate of EV20/Omomyc and HER3 has demonstrated notable anticancer effects.

Bromodomain and extra terminal BET inhibitors are among the most well-known indirect strategies. Important transcriptional regulators known as BET proteins, in particular BRD4, bind to hyperacetylated chromatin areas close to the Myc promoter to directly stimulate transcription of the Myc gene. Initial preclinical investigations demonstrated the strong efficacy of the BET inhibitor JQ1 and its co-recipients, including I-BET151 and OTX015, which effectively inhibited tumor growth and reduced Myc expression in a variety of cancer models. The outcomes of these inhibitors’ clinical studies, however, have been inconsistent. For instance, OTX015 shown a moderate level of effectiveness in treating hematological malignancies; nevertheless, phase I/II studies revealed toxicities such thrombocytopenia and gastrointestinal issues. Moreover, some cancers are resistant to BET inhibitors despite having high Myc expression, therefore the relationship between reduced Myc expression and clinical response to these drugs has not always been straightforward [107]. Protease inhibitors like MG132 and bortezomib, on the other hand, have been demonstrated to aid in the breakdown of c-Myc, lowering its activity. Additionally, these inhibitors can efficiently limit tumor development when combined with other medicines, producing synergistic benefits. PROTAC-based targeted proteolytic methods have also been suggested as a potential method of inhibiting Myc. When these substances attach to c-Myc and an E3 protease, the target protein is degraded. PROTACs can successfully lower c-Myc levels and hence block its activity, according to studies [115].

#### 6.2.2. Synthetic Lethality Approaches

This method causes cell death by simultaneously targeting two genes that have similar biological activities. Targeting compensatory mechanisms that Myc activates to deal with DNA replication stress is how this tactic works in Myc-driven malignancies. These processes include metabolic reprogramming, checkpoint-based cell cycle regulation, and DNA repair. Replication stress is increased and cancer cells are rendered artificially deadly by disrupting these mechanisms [116].

#### 6.2.3. RNA-Based Therapies

RNA-based treatments, such as mRNA vaccines, antisense oligonucleotides (ASO), and small interfering RNAs (siRNA), present viable methods for preventing virus-induced carcinogenesis and suppressing the expression of viral oncogenes. These treatments specifically target viral RNA and silence important oncogenes, including HBx in HBV or E6/E7 in HPV, to restore normal cell function. Targeting and destroying particular viral RNA, siRNA molecules attach to the complementary viral mRNA and use the RNA-induced silencing complex (RISC) to break it down. ASOs, which are short single-stranded oligonucleotides, attach to complementary RNA and either cause RNA breakdown or stop viral proteins from translating. Certain RNA molecules known as RNA aptamers attach themselves particularly to viral proteins or RNA, preventing the virus from interacting with the host cellular machinery. It is possible to train CRISPR/Cas13-based RNA editing to precisely target and cleave viral RNA, stopping the production of viral oncogenic proteins and viral replication. Additionally, shRNAs that suppress viral oncogenes or mRNA vaccines that encode viral oncogenes to trigger an immunological response in the body can be included [117]. RNA functions including transport and splicing, however, can influence the expression of c-Myc. One way to control c-Myc expression is to target the enzymes that are involved in these processes [118]. Certain circRNAs can enhance the stability of target mRNAs by interacting with RNA-binding proteins like eIF4A3.

#### 6.2.4. Preclinical and Clinical Trials

BET protein inhibitors’ therapeutic efficacy in AML preclinical and clinical trials may support the viability of c-Myc expression targeting by modifying Myc transcriptional activation. Even though further clinical research is needed, the encouraging results of BET inhibitors in both monotherapy and combination therapy support the strategy of blocking signal transduction in the constantly changing field of AML treatment. Cyclin D1, MCL-1, and BCL-2 levels were reduced by alvocidib in AML patient samples. Alvocidib showed synergy with conventional therapy comprised of 7 + 3 cytarabine and mitoxantrone in a phase II clinical investigation of adult AML patients with poor prognoses. For newly diagnosed secondary AML, the 75% complete response (CR) rate and the first relapse following a brief CR were both noticeably greater than those of conventional 7 + 3 therapy. It has been demonstrated that voruciclib, another CDK9 inhibitor, lowers the amount of c-Myc expression in AML cell lines and patient samples. In a phase I clinical trial, voruciclib is now being evaluated for safety and tolerability in the treatment of B-cell malignancies and AML. A phase I clinical research involving individuals with R/R AML investigated buparlisib, which has shown efficacy and extended survival in mouse models [106]. Gedatolisib suppressed PI3K/AKT/mTOR signaling and shown anti-neoplastic efficacy in solid tumor xenograft models [119]. Everolimus plus chemotherapy were assessed for clinical efficacy and tolerability in AML patients under 65 years of age in a phase Ib trial. A decrease in the phospho-P70S6 kinase product suggested that everolimus administration successfully inhibited mTORC1 signaling. Furthermore, with a weekly dosage of medication, 19 out of 28 patients experienced total remission [120]. A summary of selected clinical trials targeting c-Myc directly or indirectly is presented in Table 2.

## 7. Role of c-Myc in Treatment Resistance

### 7.1. Resistance Mechanisms Involving c-Myc

The transcription factor Myc promotes cancer cells’ resistance to treatment and controls a number of processes. Excess Myc makes cancer cells more susceptible to oxidative phosphorylation inhibitors in addition to making them resistant to treatment. Additionally, Myc can phosphorylate transcription factors, promote the expression of stress-resistant genes, and increase cell survival via activating the integrated stress response (ISR)/unfolded protein response (UPR) pathway while blocking the translation of elF2α. Research has demonstrated that mutations in the Myc-destroying tumor suppressor gene FBXW7 also result in resistance to certain chemotherapy medications [88]. The JAK2 pathway is active during the STAT signaling pathway, and it phosphorylates one of the STAT members in response to cytokines released by cancer cells. Once this protein has been phosphorylated, it travels to the nucleus and controls the transcription of several genes, including c-Myc. By prolonging the cell cycle and generating ROS, CML contributes to the advancement of the disease through the STAT5 pathway and prevents apoptosis. In order to avoid BCR-ABL1 suppression, CML cells also initiate and activate alternative pathways, including JAK, SRC, RAS/MAPK, WNT/β-catenin, STAT, and PI3K/AKT. When carcinogenic pathways are transferred to earlier pathways, apoptosis is inhibited and survival is increased. Myc is a crucial transcription factor for cell survival among others [87]. The enrichment of oxidative phosphorylation pathways, mitochondrial biogenesis, and anti-apoptotic gene profiles were also linked to Myc-induced resistance, according to transcriptomic analysis. Research demonstrates that in vitro, resistant AML cells were sensitised by pharmacological suppression of Myc using F4-10058. Also, Myc, a key player in metabolic programming, decreases apoptosis, which makes AML resistant to treatment [121]. It should be noted that c-Myc pathway and the apoptotic propensity are triggered when KLF4 is inhibited. Upregulation of c-Myc normally prevents LSCs from surviving and functioning, however in leukemia, cells improve survival by changing KLF4 and blocking p53. The BCR-ABL1 oncoprotein promotes Myc expression and a favorable cycle for the advancement of illness by blocking PPA2. Moreover, across the WNT/β-catenin pathway. A major contributing element to medication resistance is the ongoing activation of c-Myc. Additionally, Myc is a key target of the PPA2 pathway, and inhibiting it is akin to causing drug resistance. Chromosomal defects cause cell proliferation by suppressing the ubiquitin ligase FBXW7, which in turn stimulates Myc expression [122,123]. BCR-ABL suppresses BAX activation via multiple routes, including elevated BCL-XL expression, and the c-Myc protein is linked to anti-apoptotic actions. By stopping BAX from being translocated to mitochondria, BCL-XL can stop apoptosis [124].

According to recent research, chemo sensitivity is linked to an enhancer domain that regulates metabolic characteristics in human LSCs. It should be mentioned that the BET family proteins that control Myc expression and the BRD4 protein of the same family are thought to be novel therapeutic targets in AML. Additionally, because BET and BRD4 control Myc expression, resistance to BET inhibitors indicates that the c-Myc suppression pathway is not working [125].

### 7.2. Implications for Therapy Efficacy

IRAK4 inhibitors and CELMoDs, particularly GSPT1 degraders like CC-885 or CC-90009, can work in concert to improve the therapeutic outcome for AML, according to the study. Long-term pretreatment with an IRAK4 inhibitor increases the sensitivity of AML cells to CC-885. Their IC50 falls, in other words. Following IRAK4 pretreatment, patient samples also showed the similar pattern of elevated sensitivity to CC-885. More significantly, normal CD34+ hematopoietic cells showed far less of this combination effect, indicating that ordinary cells are less susceptible to the deadly effects of combination therapy than cancerous cells. Additionally, clonogenic tests have demonstrated that while IRAK4 or CC-885 when used alone have just a slight effect, when used in combination, they very efficiently prevent cells from forming colonies. Lastly, apoptosis is induced by c-Myc’s abrupt decrease below a critical threshold, which increases the effectiveness of therapy because it is recognized to be a crucial oncoprotein for AML cell proliferation and survival [121]. In several models of AML, research has also demonstrated a considerable synergistic impact of Selinexor and JQ1. The two medications had a genuine synergistic effect when the Combination Index was less than 1 and the HSA score was greater than 10. Each medication was less successful when used alone, but in cell studies, the combination suppressed almost 80% of leukemia cells. In both animal and PDX models, the combination treatment greatly improved animal longevity while reducing the tumor burden in the liver, spleen, and bone marrow. The combination of the two medications also shown a higher cytotoxic effect than either treatment alone and considerably decreased c-Myc expression in early patient samples. The results further shown that the combination increases treatment efficacy by dual-inhibiting c-Myc at the mRNA and protein levels. Effectively blocking c-Myc as a primary target may therefore be a novel way to increase the effectiveness of AML treatment and get past medication resistance [126]. AML cell and animal models demonstrated a notable synergistic impact when chidamide and cytarabine were combined. By blocking the Myc signaling pathway and binding directly with the c-Myc protein, the combination decreased the expression of the RRP9 gene. Reduced rRNA synthesis, decreased ribosome biogenesis, and decreased recombinant protein production were all consequences of the decrease in RRP9 levels. Both MV4-11 and Kasumi-1 cell models showed these effects, and early patient samples corroborated them. In in vivo studies, the combined therapy improved mouse survival and decreased tumor burden [127]. According to the results, forskolin may have a new way of working and may be used in conjunction with the common medication daunorubicin to treat AML with KMT2A rearrangements. In addition to causing cell death and inhibiting cell proliferation, forskolin also alters the expression of the genes Myc, HOXA9, and HOXA10, making leukemia cells more susceptible to daunorubicin. Forskolin’s cytotoxic effect is partially mitigated by silencing the PPP2CA gene, which also activates certain signaling pathways like ERK1/2 and inhibits GSK3β. However, this mechanism is not responsible for the increased effect of forskolin on daunorubicin sensitivity, which results from increased intracellular accumulation of daunorubicin due to inhibition of the P-glycoprotein 1 drug efflux pump in leukemia cells. Accordingly, the findings imply that forskolin and the common medication daunorubicin may improve treatment outcomes, especially in AML with KMT2A rearrangements, and enable the use of lower dosages of chemotherapeutic medications to minimize adverse effects [128].

### 7.3. Current Issues and Therapeutic Modalities

#### 7.3.1. Radiotherapy: Role and Limitations in Leukemia Treatment

One of the most successful leukemia treatment modalities is radiotherapy, which is used to treat both primary and terminal tumors. Chemotherapy is effective on high-sensitivity tumors because of the steep response curve that these tumors display [129]. High power and energy ionizing radiation kills the components inside brain cells and kills cancer cells. Note that there are drawbacks to radiation therapy, including dose variability, innate radiation resistance, and uncommon tumor recurrence. Today, this approach is delivered and transformed into a targeted and specialized therapeutic procedure thanks to the addition of nanoparticles. By causing processes including reactive oxygen species generation, DNA damage, suppression of the DNA repair system, and cell cycle arrest, radiation treatment based on nanoparticles kills cancer cells. Transplanting allergen hematopoietic stem cells is another of the most useful therapeutic approaches. This approach lowers the risk of disease development by up to 50% through strong cytoreduction in chemotherapy, radiation therapy, or the immunological response known as graft-versus-leukemia (GVL) [130]. Radiation therapy for cancer now employs innovative therapeutic modalities, such as whole-body radiotherapy (TBI), total bone marrow radiotherapy (TMI), and intraoperative radiotherapy. Because it delivers high doses directly to the bone marrow, the TMI approach is utilized in patients undergoing bone marrow transplantation (BMT), while the volumetric modulated technique is employed in whole-body radiotherapy to minimize organ toxicity. Both cranial and whole-body irradiation are used to prepare for bone marrow transplantation by irradiating the entire body with a consistent amount of radiation [131,132]. Limitations of radiation therapy in general include systemic toxicity and kidney, liver, and lung damage. Additionally, the dose needs to be carefully regulated, and the TMI approach requires sophisticated equipment and differs from the TBI method in a few ways. It is important to remember, however, that the majority of research are still in the clinical stage and have not yet produced conclusive findings [131].

#### 7.3.2. Chemotherapy: c-Myc’s Influence on Chemo Sensitivity and Resistance

In leukemia cells, c-Myc overexpression directly contributes to chemoresistance through multiple mechanisms. c-Myc-driven upregulation of anti-apoptotic BCL-2 family members, particularly BCL-2 and MCL-1, raises the apoptotic threshold, rendering leukemia cells less responsive to genotoxic agents [40]. Additionally, c-Myc enhances the expression of drug efflux transporters, promoting the active extrusion of chemotherapeutic agents, and activates DNA damage repair pathways that counteract chemotherapy-induced cytotoxicity [48]. In AML, c-Myc-mediated metabolic reprogramming toward oxidative phosphorylation and glutaminolysis has been linked to resistance against cytarabine and anthracyclines [121]. Furthermore, c-Myc stabilization through FBXW7 mutations or PLK1-mediated phosphorylation directly impairs the apoptotic response to chemotherapy in T-ALL and AML [38,45].

Notably, telomerase inhibition has been shown to induce apoptosis in AML stem cells through the activation of both intrinsic and extrinsic apoptotic pathways, representing a promising strategy to eliminate the LSC population that is frequently responsible for relapse and chemo-resistance [133].

#### 7.3.3. Immunotherapy: Interaction Between c-Myc Signaling and Immune Responses

**Figure 2:**
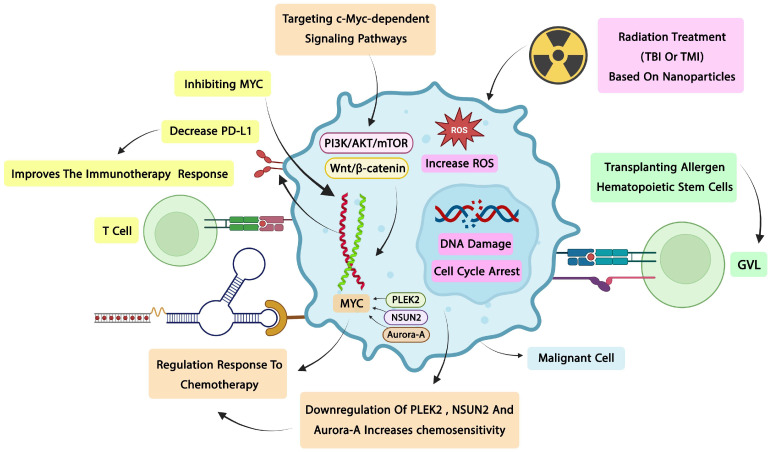
Diagrammatic depiction of leukemia targeting techniques and c-Myc-driven mechanisms of treatment resistance. This diagram highlights the crucial function of c-Myc in coordinating transcriptional, metabolic, and survival pathways that together lead to resistance to immunotherapy, chemotherapy, and radiation. Through persistent c-Myc expression, dysregulated activation of upstream signaling networks, including as PI3K/AKT/mTOR, WNT/β-catenin, JAK/STAT, and BCR-ABL–associated cascades, increases mitochondrial activity, modulates reactive oxygen species, improves DNA-damage tolerance, and evades apoptosis. PLEK2, NSUN2, and Aurora-A are examples of downstream mediators that support leukemic cell persistence under therapeutic pressure by further stabilizing c-Myc and strengthening feed-forward loops. Myc-directed pharmacologic inhibition, BET protein blockade, Aurora-A suppression, nanoparticle-based radiotherapy, IRAK4 inhibition in conjunction with GSPT1 degraders, and graft-versus-leukemia effects after hematopoietic stem-cell transplantation are among the new interventions highlighted in the diagram that are intended to disrupt these resistance circuits. Combinatorial treatment approaches that target both Myc signaling and its auxiliary survival pathways are justified by these components, which together show c-Myc as a major hub of resistance. Created in BioRender. Keyhani, A. https://BioRender.com/zegyk6o (accessed on 16 May 2026). Agreement number: YY29Q13K8Q.

ASPH-Myc causes tumor cells to express more PD-L1 and activates the NOTCH pathway. T cell exhaustion and malfunction, as well as tumor escape from the immune system, are caused by the rise in these molecules [134] (Figure 2). Acute myeloid leukemia (AML) and chronic lymphocytic leukemia (CLL) are linked to overexpression of Myc, which also inhibits the production of the Period2 gene, is overexpressed in neutrophils, and increases antitumor proliferation. Directly or indirectly, c-Myc-induced signaling molecule dysregulation can aid tumors in evading the immune response. For instance, Myc can attach to the promoter of CD47, which binds to SIRPα and phosphorylates ATM, which inactivates myosin. Tumor growth and phagocytosis inhibition are the results of all these impacts. In this sense, EPCAM is a key marker for cancer stem cells, and Myc plays a significant role in controlling the activity of cancer stem cells. When EPCAM is cleaved, the intracellular domain contributes to the transcription complex and triggers the production of cyclins A and E as well as c-Myc [135]. An investigation into the role of Myc in leukemia and lymphoma tumors using transgenic mouse models revealed that inactivating Myc in leukemia and lymphoma cells inhibited tumor growth, a process that is reliant on the host immune system. According to earlier research, Myc can influence innate immunity by controlling MHC class 1. Restoring the immune response against tumors, particularly in leukemia, is another novel treatment approach that focusses on the Myc pathway [136].

Since MDSCs is produced by increased c-Myc expression in EV external vesicles, addressing this process can impact the efficacy of immunotherapy. This mechanism is thought to be a means of evading the immune system. The tumor suppressor microenvironment includes MDSCs, hence this approach has been suggested as a way to get beyond the immune system. Since cytotoxic T cells, the target of various immunotherapies, are inhibited in this environment, they may be used as a supplement to enhance the immunotherapy procedure [36,137]. Additionally, radio immunotherapy is a type of targeted radiation therapy that delivers ionizing radiation to the tumor cell by using monoclonal antibodies or their fragments as carriers [129]. CAR-NK cell therapy has emerged as a promising immunotherapeutic strategy for AML, offering advantages over CAR-T approaches, including a reduced risk of cytokine release syndrome and the potential for allogeneic use [138].

#### 7.3.4. Challenges in Clinical Implementation

Presently, leukemia treatment faces obstacles such medication side effects, patient non-adherence to treatment, and financial hardships. It should be mentioned that using BKTs for therapy carries a higher risk of bleeding, cardiovascular problems, and infection, among other clinical difficulties and medication side effects. PI3K inhibitors may also result in cytopenia, hepatotoxicity, infection, colitis, pneumonitis, and allergic skin responses. The high expense of medications is another issue that might be brought up in relation to treatment adherence, as are issues like issues with the treatment plan and a phobia of utilizing pharmaceuticals. It is also crucial to address issues in the health system such inadequate care coordination, a lack of social support, treatment delays, and even a failure to manage drug interactions [139].

Diagnostic difficulties including unequal access to treatment and advanced technologies, as well as a lack of health infrastructure, can be considered a significant problem in many nations. Clinical heterogeneity is a major problem in the modern period since it is hard to predict which individuals will have an aggressive course and which will have an indolent one, and treatment decisions are made more complex when numerous indicators, like Myc abnormalities, are present [140,141].

The integration of omics data, the heterogeneity of epigenetic markers, radiation resistance, immunotherapy failure, and even metastasis can be significant problems in this area. According to recent research, the most significant obstacles that need more study and focus are the requirement for live cells for culture, intricate in vitro procedures, FISH limitations, high RNA-Seq costs, the inability to create fusion transcripts at the RNA level, and the requirement for multiple samples [142]. Sometimes TNGS at the DNA level only detects the reciprocal form, while the original form can only be detected at the RNA level, which is a limitation at the DNA level. Additionally, some mutations at the DNA level do not result in the formation of fusion transcripts on RNA, causing conflicts between different tests. Reviews have proposed epigenetic drug recovery tactics, the application of artificial intelligence, and the integration of omics data as current answers to these problems [143].

## 8. Future Perspectives

### 8.1. Personalized Medicine Approaches

Additional research is required to assess the TP53 mutational status and its correlation with Myc anomalies in order to more precisely identify the prognosis of high-risk categories. Even with the progress made with targeted medicines, prospective trials are still needed to validate the incorporation of genetic risk factors like CLL-IPI into the treatment of patients receiving new drugs. Current research will shed light on the possibilities of time-limited treatments, including combinations of obinutuzumab, ibrutinib, and venetoclax in high-risk patients with del17p/TP53 mutation [144]. Because of its intricacy, CLL necessitates a customized strategy in which treatment plans are based on each patient’s unique genetic profile. Treatment choices are based on the IGHV and TP53 mutation status, since individuals with TP53 mutations typically respond better to targeted medicines than chemo immunotherapy. Treatment decisions for individuals who have relapsed or are resistant are influenced by genetic characteristics such as del17p/TP53mut and IGHV status, as well as prior medications [145]. Extending these ideas, biomarkers are important instruments in personalized medicine that provide benefits for prognosis, early diagnosis, and therapy response prediction. They make it possible to choose suitable patients for treatment and to give the proper drug to the right patient. Continuous improvements in biomarker identification and the creation of therapeutic approaches tailored to each patient’s drug and dosage requirements are necessary to meet these goals. The major objective is still to provide individually tailored patient therapy, but future directions include improving biomarker discovery, lowering drug development costs, and using next-generation sequencing testing to increase treatment efficacy and precision [146]. Using these ideas as a foundation, new therapeutic approaches for hematological malignancies, like BH3 mimetics and targeted inhibitors of particular signaling pathways, are promising for improving tailored treatments. T-cell acute lymphoblastic leukemia (T-ALL) may benefit from Notch pathway inhibitors, according to recent research, which shows that patient-specific pathway targeting can increase therapeutic effectiveness while reducing side effects. Furthermore, precise patient categorization and combination therapy optimization are made possible by the identification of additional biomarkers made possible by the integration of next-generation sequencing and other molecular profiling methods [147].

### 8.2. Novel Delivery Systems

Research on colon cancer cells has shown a new mechanism involving the degradation of c-Myc and the endoplasmic reticulum. This technique uses NK-1R antagonists to trigger apoptosis by causing stress in the endoplasmic reticulum. This route leads to the release of calcium and the destruction of the c-Myc protein [148]. Compared to chemotherapy, this new mechanism has increased the cellular response. It should be mentioned that novel approaches to drug delivery are also being employed, such as the use of titanium nanoparticles, which build up in tumors and make it easier for drugs to reach their target location. These medications have a higher bioavailability, a longer half-life, and the capacity to accumulate more in the tumor [61].

The PROTAC proteolysis targeting chimaera, which uses a ligand for the target protein along with an adaptor and linker to engage E3 ligase, is another novel technology in use today. Together with an E3 ligase, proteac binds to its target protein, causing proteasome destruction. Since many Myc inhibitor molecules do not readily cross the cell membrane, one of the primary challenges in developing a suitable method for drug delivery to patients is overcoming the barrier to therapeutic resistance and increasing selectivity. This is one of the key benefits of the novel drug system. Using a cell-penetrating peptide sequence in the CPP is one of the popular methods for getting peptides into living cells [149,150].

It has also been suggested to use modified exosomes. Due to their inherent biocompatibility, these exosomes can target and penetrate a variety of obstacles, making them a possible solution to these problems. Overall, restrictions including drug resistance, molecular, systemic toxicity, drug fragility, survival of cancer stem cells, and their adverse effects underscore the need for novel therapeutic strategies [151].Telomerase-based therapeutic strategies have shown considerable promise in hematological malignancies, with approaches including telomerase inhibitors, telomerase-targeted immunotherapy, and telomere-disrupting agents demonstrating preclinical efficacy across leukemia subtypes [152].

### 8.3. Potential for Combination Therapies

By boosting cleaved PARP, caspase-9, and caspase-3 and decreasing BCL2 and MCL-1, the combination of cladribine (CLA) with venetoclax (VEN) improves anti-leukemic efficacy and overcomes VEN resistance in AML cells. The therapy suppresses both c-Myc and DNA-PKcs, and inhibiting DNA-PKcs further reduces the expression of c-Myc and inhibits its growth. Elevated levels of DNA-PKcs in AML patients and their associated with poor outcomes, along with a positive correlation between DNA-PKcs and c-Myc expression, suggest the clinical promise of the venetoclax and cladribine combination in AML [153].

Additionally, XPO1 suppression results in nuclear retention of eIF4E, which lowers c-Myc expression and AML cell survival. Concurrent downregulation of eIF4E and XPO1 further inhibits c-Myc and promotes apoptosis. A poor prognosis is linked to the increased expression of XPO1 and eIF4E in AML patients, indicating that targeting both of these proteins together may be beneficial for treating AML [43].

When CpG 685 and imatinib are combined, Sup-B15 resistance to either CpG 685 or imatinib alone is reversed. In resistant Sup-B15 cells, imatinib activated BAX and increased sensitivity to CpG 685 by blocking BCR-ABL signaling and downregulating c-Myc and BCL-XL expression. Imatinib and CpG 685 combined therapy extended the lifespan of Sup-B15 cell engraftment in NCG mouse xenograft models in vivo. When combined with imatinib, CpG 685 further decreased Sup-B15 cells in mouse bone marrow, spleen, and peripheral blood, even in cases where it was ineffective on its alone. One of the main obstacles to treating B-ALL is medication resistance, so using CpG ODNs in conjunction with targeted medicines may be a potential therapeutic approach [124].

Beyond these specific combinations, several broader therapeutic frameworks warrant further investigation. The combination of BET bromodomain inhibitors with CDK9 inhibitors achieves deeper Myc suppression by concurrently blocking transcriptional initiation and elongation, and has demonstrated synergistic anti-leukemic activity in preclinical AML models [38]. Similarly, combining Myc-directed therapies with immune checkpoint blockade represents a particularly compelling but underexplored strategy. Given that c-Myc transcriptionally upregulates PD-L1 and CD47 on leukemia cells [23,24], pharmacological suppression of c-Myc may reduce immune checkpoint ligand expression, thereby sensitizing leukemic cells to anti-PD-1/PD-L1 antibodies and enhancing CAR-T cell efficacy [128]. In CLL models, the c-Myc inhibitor 10058-F4 has been shown to decrease PD-L1 surface expression and restore T-cell function [59].

The emergence of proteolysis-targeting chimeras (PROTACs) that recruit E3 ubiquitin ligases to c-Myc represents a revolutionary approach to bypass the undruggable nature of the c-Myc protein [149]. Combining Myc-targeting PROTACs with conventional chemotherapeutic agents such as cytarabine or daunorubicin may prevent the Myc-driven transcriptional recovery that occurs following DNA damage, potentially converting transient cytostatic responses into durable remissions [112]. Additionally, epigenetic priming with HDAC inhibitors can reverse repressive chromatin states at interferon-responsive gene loci, restoring antitumor immunity while simultaneously suppressing Myc transcription [43]. Sequential or concurrent administration of HDAC inhibitors with agents that block Myc protein stability, such as Aurora kinase inhibitors, may create a synthetic lethal interaction in leukemia cells [40]. Future clinical trials should prioritize biomarker-stratified combination regimens that incorporate quantitative measurements of Myc pathway activity to prospectively identify patients most likely to benefit from these combined approaches.

## 9. Conclusions

Dysregulation of the c-Myc oncogene, a master regulator of cell survival, metabolism, and proliferation, is essential for the development and spread of leukemia. Atypical activation of the XPO1/eIF4E/c-Myc axis promotes cancer cell survival in acute myeloid leukaemia (AML). It has been demonstrated that the XPO1 inhibitor Selinexor (KPT-330) and the hypo ethylating drug Azacitidine work in concert to limit AML cell growth and trigger apoptosis by downregulating the levels of XPO1, eIF4E, and most significantly, the c-Myc protein. A poor prognosis is correlated with elevated expression of XPO1 and eIF4E in AML patients, highlighting the therapeutic utility of focusing on this regulatory mechanism. The carcinogenic persistence of c-Myc is mostly due to post-translational stabilization, which goes beyond transcriptional control. Excessive buildup and persistent malignant signaling result from kinase dysfunctions that impair normal Myc degradation, including in Aurora kinase A and Polo-like kinase 1 (PLK1). By targeting these kinases, Myc levels in leukemic cells are selectively decreased while normal cells are left unaffected. Moreover, c-Myc dysregulation is significantly influenced by epigenetic processes. Myc-responsive gene transcription and chromatin accessibility are regulated by histone deacetylases (HDACs). HDAC inhibition reactivates antitumor immunity by boosting interferon pathways and immune cell infiltration, and it reduces Myc-driven oncogenic signaling, particularly when combined with other treatments. Additionally, the idea of synthetic lethality, which holds that cells depending on Myc rely on auxiliary survival pathways, provides an extra treatment approach because leukemia cell death results from the simultaneous blockage of Myc and these pathways.

Additionally, c-Myc contributes to immune suppression in leukemia by directly inducing the expression of immune checkpoint molecules like PD-L1 and CD47. Several oncogenic signaling pathways, such as WNT/β-catenin, IL-6/JAK2/STAT3, MAPK, and PI3K/AKT/mTOR, influence c-Myc expression. Dysregulation of WNT signaling promotes nuclear accumulation of β-catenin, which enhances Myc transcription; IL-6-mediated activation of JAK2/STAT3 not only increases Myc expression but also drives metabolic reprogramming through the upregulation of GLUT1 and LDHA; under cellular stress, PKM2 facilitates IRES-mediated Myc translation, which reinforces glutamine metabolism; RNA-binding proteins like DDX6 further stimulate glycolysis and proliferation; on the other hand, miR-124 downregulates Myc, suppressing glycolysis.

Immune escape is made possible in chronic lymphocytic leukemia (CLL) by the NOTCH–c-Myc axis, which stimulates PD-L1 expression. Additionally, c-Myc suppresses the microRNAs miR-26a and miR-101, which raises EZH2. This, in turn, improves histone methylation and maintains the transcription of Myc and PD-L1 in a positive feedback loop. Immune sensitivity is restored by pharmacologic Myc inhibitors like 10058-F4, which efficiently lower Myc and PD-L1 expression. Elevated c-Myc levels functionally promote leukemic transformation, apoptosis resistance, and fast proliferation. Increased FLT3 and STAT3 activity and decreased p27 are correlated with c-Myc overexpression in AML, which promotes unchecked development. Leukemic growth is accelerated in ALL when the transcription factor IKAROS (IKZF1) is lost because it eliminates inhibitory control over Myc transcription. Similarly, c-Myc expression and downstream targets (Cyclin D1, MMP7) are enhanced by HBO1-mediated stimulation of WNT/β-catenin signaling, maintaining survival and proliferation. All things considered, in leukemia, c-Myc serves as a key mediator between immunological control, epigenetic modification, and oncogenic signaling. Myc-targeted treatments, either directly or through upstream/downstream pathway inhibition, are promising ways to overcome drug resistance and enhance patient outcomes because of its complex function in leukemogenesis.

## Figures and Tables

**Table 1:** Mechanisms and Functional Consequences of c-Myc Dysregulation Across Leukemia Subtypes.

Leukemia Subtype	Mechanism of c-Myc Dysregulation	Key Molecular Mediators	Functional Consequences	Clinical Impact	Refs.
AML	Transcriptional activation, protein stabilization, impaired degradation	FLT3-ITD → STAT3; XPO1/eIF4E axis; PLK1/AURKA stabilization; FBXW7 mutations	Enhanced proliferation, metabolic reprogramming (glycolysis, glutaminolysis), LSC maintenance, apoptosis resistance	Adverse prognosis, chemotherapy resistance, poor overall survival	[37,43,45,67]
B-ALL	Loss of transcriptional repression, epigenetic activation	IKZF1 deletion → loss of c-Myc repression; HBO1 → H3K14ac → WNT/β-catenin activation; PAK4-mediated phosphorylation (S67)	Blocked B-cell differentiation, uncontrolled proliferation, MCL-1-mediated survival	High-risk disease, inferior outcomes, relapse propensity	[70,71,74]
T-ALL	NOTCH1-Myc synergy, impaired degradation	NOTCH1 activation → Myc transcription; FBXW7 loss-of-function mutations; TAL1/AURKB feedforward loop	Rapid cell cycle progression, enhanced anabolic metabolism, resistance to γ-secretase inhibitors	Aggressive clinical course, therapeutic challenges	[37,38]
CML	BCR-ABL1 downstream signaling	BCR-ABL1 → PI3K/AKT/mTOR, JAK/STAT5, RAS/MAPK → Myc transcription; PP2A inhibition → Myc stabilization	Disease progression, genomic instability, blast crisis transition	TKI resistance, disease transformation	[87]
CLL	BCR signaling, NOTCH-Myc axis, protein stabilization	PKCα-KR → Myc S62 phosphorylation; NOTCH → Myc → EZH2 positive feedback; IGH enhancer remodeling	Immune evasion (PD-L1, CD47 upregulation), apoptosis resistance, T-cell dysfunction	Poor prognosis, immunotherapy resistance	[68,88]

**Table 2:** Selected Completed and Ongoing Clinical Trials Targeting c-Myc Directly or Indirectly in Leukemia.

Therapeutic Agent	Target/Mechanism	Leukemia Subtype	Phase	Key Findings/Outcomes	Refs.
Selinexor (KPT-330)	XPO1 inhibitor → nuclear retention of eIF4E → reduced c-Myc translation	R/R AML, MDS	Phase I/II	Synergy with azacitidine; reduced XPO1/eIF4E/c-Myc; improved response in combination	[43]
Alvocidib (Flavopiridol)	CDK9 inhibitor → transcriptional repression of Myc, MCL-1	Newly diagnosed secondary AML, R/R AML	Phase II	75% CR with 7 + 3 regimen in newly diagnosed; MCL-1 downregulation confirmed	[106]
Voruciclib	CDK9 inhibitor → reduced c-Myc and MCL-1 expression	R/R AML, B-cell malignancies	Phase I	Ongoing; safety and tolerability evaluation; c-Myc target engagement demonstrated preclinically	[109]
JQ1/OTX015	BET bromodomain inhibitor → displacement of BRD4 from Myc locus	AML, ALL, CLL	Phase I/II	Moderate efficacy in monotherapy; thrombocytopenia and GI toxicities dose-limiting; potential for combination strategies	[107]
Buparlisib	PI3K inhibitor → downstream Myc suppression	R/R AML	Phase I	Efficacy and prolonged survival in preclinical models; clinical evaluation ongoing	[106]
Everolimus + Chemotherapy	mTORC1 inhibitor → reduced Myc translation	AML (<65 years)	Phase Ib	mTORC1 inhibition confirmed (↓phospho-P70S6K); 19/28 patients achieved CR with weekly dosing	[120]

## Data Availability

No new data were created or analyzed in this study.

## References

[1] Chennamadhavuni A., Lyengar V., Mukkamalla S., Shimanovsky A. (2023). Leukemia. StatPearls.

[2] Lu J., Zhang Y., Wang S., Bi Y., Huang T., Luo X., Cai Y.-D. (2020). Analysis of four types of leukemia using gene ontology term and Kyoto encyclopedia of genes and genomes pathway enrichment scores. Comb. Chem. High Throughput Screen..

[3] Liu Y., Yang Z., Zhang Q., Hai P., Zheng Y., Zhang J., Pan X. (2025). Recent advances in signaling pathways and kinase inhibitors for leukemia chemotherapy. Curr. Med. Chem..

[4] Daltveit D.S., Morgan E., Colombet M., Steliarova-Foucher E., Bendahhou K., Marcos-Gragera R., Rongshou Z., Smith A., Wei H., Soerjomataram I. (2025). Global patterns of leukemia by subtype, age, and sex in 185 countries in 2022. Leukemia.

[5] Tsilingiris D., Vallianou N.G., Spyrou N., Kounatidis D., Christodoulatos G.S., Karampela I., Dalamaga M. (2024). Obesity and leukemia: Biological mechanisms, perspectives, and challenges. Curr. Obes. Rep..

[6] Liao Y., Zhong L., Zhao Y., Wan P., Zhang Y., Deng Y., Zhang H., Wang M., Liu B. (2025). OTUB1 promotes the progression of acute myeloid leukemia by regulating glycolysis via deubiquitinating c-Myc. Cell. Signal..

[7] Venugopal S., Sekeres M.A. (2024). Contemporary management of acute myeloid leukemia: A review. JAMA Oncol..

[8] Sharma V., Kushwaha V., Panwar A., Sheikh I., Anuprabha M.S., Upadhyay S.K., Ramniwas S., Arora P., Sharma A.K. (2024). Oncogenes and oncogenesis: Origin, development, cause, and therapy. Anticancer Therapeutics: Bionanotechnology, Nanomedicine and Phytochemicals.

[9] Illi B., Nasi S. (2023). Myc beyond cancer: Regulation of mammalian tissue regeneration. Pathophysiology.

[10] Travaglini S., Gurnari C., Ottone T., Voso M.T. (2024). Advances in the pathogenesis of FLT3-mutated acute myeloid leukemia and targeted treatments. Curr. Opin. Oncol..

[11] El-Tanani M., Nsairat H., Matalka I.I., Lee Y.F., Rizzo M., Aljabali A.A., Mishra V., Mishra Y., Hromić-Jahjefendić A., Tambuwala M.M. (2024). The impact of the BCR-ABL oncogene in the pathology and treatment of chronic myeloid leukemia. Pathol.-Res. Pract..

[12] Ahmad F., Shah A., Angi M., Narmawala Q., Gupta I., Chaudhary P., Jajodia E., Vaishnani T., Manguika N., Haque M. (2024). Identification of a novel cryptic variant chromosomal rearrangement involving 9q34, 22q11. 2, and 5q22 resulting in ins (9; 22) and t (5; 22) in chronic myeloid leukemia: A case report. Ann. Hematol..

[13] Rondoni M., Marconi G., Nicoletti A., Giannini B., Zuffa E., Giannini M.B., Mianulli A., Norata M., Monaco F., Zaccheo I. (2025). Low WT1 Expression Identifies a Subset of Acute Myeloid Leukemia with a Distinct Genotype. Cancers.

[14] Baranwal A., Basmaci R., He R., Viswanatha D., Greipp P., Murthy H.S., Foran J., Palmer J., Hogan W.J., Litzow M.R. (2024). Genetic features and outcomes of allogeneic transplantation in patients with WT1-mutated myeloid neoplasms. Blood Adv..

[15] Yan X.Y., Kang Y.Y., Zhang Z.Y., Huang P., Yang C., Naranmandura H. (2024). Therapeutic approaches targeting oncogenic proteins in myeloid leukemia: Challenges and perspectives. Expert Opin. Ther. Targets.

[16] Das S.K., Lewis B.A., Levens D. (2023). MYC: A complex problem. Trends Cell Biol..

[17] Neel B.G., Jhanwar S.C., Chaganti R., Hayward W.S. (1982). Two human c-onc genes are located on the long arm of chromosome 8. Proc. Natl. Acad. Sci. USA.

[18] Dalla-Favera R., Bregni M., Erikson J., Patterson D., Gallo R.C., Croce C.M. (1982). Human c-myc onc gene is located on the region of chromosome 8 that is translocated in Burkitt lymphoma cells. Proc. Natl. Acad. Sci. USA.

[19] Wasylishen A.R., Penn L.Z. (2010). Myc: The beauty and the beast. Genes Cancer.

[20] Taub R., Moulding C., Battey J., Murphy W., Vasicek T., Lenoir G.M., Leder P. (1984). Activation and somatic mutation of the translocated c-myc gene in Burkitt lymphoma cells. Cell.

[21] Deng S., Chen B., Huo J., Liu X. (2022). Therapeutic potential of NR4A1 in cancer: Focus on metabolism. Front. Oncol..

[22] Schaub F.X., Dhankani V., Berger A.C., Trivedi M., Richardson A.B., Shaw R., Zhao W., Zhang X., Ventura A., Liu Y. (2018). Pan-cancer alterations of the MYC oncogene and its proximal network across the cancer genome atlas. Cell Syst..

[23] Kim E.Y., Kim A., Kim S.K., Chang Y.S. (2017). MYC expression correlates with PD-L1 expression in non-small cell lung cancer. Lung Cancer.

[24] Casey S.C., Tong L., Li Y., Do R., Walz S., Fitzgerald K.N., Gouw A.M., Baylot V., Gütgemann I., Eilers M. (2016). MYC regulates the antitumor immune response through CD47 and PD-L1. Science.

[25] Burger J.A., Chiorazzi N. (2013). B cell receptor signaling in chronic lymphocytic leukemia. Trends Immunol..

[26] Trejo-Solís C., Castillo-Rodríguez R.A., Serrano-García N., Silva-Adaya D., Vargas-Cruz S., Chávez-Cortéz E.G., Gallardo-Pérez J.C., Zavala-Vega S., Cruz-Salgado A., Magaña-Maldonado R. (2024). Metabolic roles of HIF1, c-Myc, and p53 in glioma cells. Metabolites.

[27] Larochelle S., Amat R., Glover-Cutter K., Sansó M., Zhang C., Allen J.J., Shokat K.M., Bentley D.L., Fisher R.P. (2012). Cyclin-dependent kinase control of the initiation-to-elongation switch of RNA polymerase II. Nat. Struct. Mol. Biol..

[28] Nair S.K., Burley S.K. (2003). X-ray structures of Myc-Max and Mad-Max recognizing DNA: Molecular bases of regulation by proto-oncogenic transcription factors. Cell.

[29] Beaulieu M.-E., Castillo F., Soucek L. (2020). Structural and Biophysical Insights into the Function of the Intrinsically Disordered Myc Oncoprotein. Cells.

[30] Chen H., Liu H., Qing G. (2018). Targeting oncogenic Myc as a strategy for cancer treatment. Signal Transduct. Target. Ther..

[31] Mateyak M.K., Obaya A.J., Adachi S., Sedivy J.M. (1997). Phenotypes of c-Myc-deficient rat fibroblasts isolated by targeted homologous recombination. Cell Growth Differ..

[32] Steiner P., Philipp A., Lukas J., Godden-Kent D., Pagano M., Mittnacht S., Bartek J., Eilers M. (1995). Identification of a Myc-dependent step during the formation of active G1 cyclin-cdk complexes. EMBO J..

[33] Schmid P., Schulz W.A., Hameister H. (1989). Dynamic expression pattern of the myc protooncogene in midgestation mouse embryos. Science.

[34] Hirvonen H., Mäkelä T., Sandberg M., Kalimo H., Vuorio E., Alitalo K. (1990). Expression of the myc proto-oncogenes in developing human fetal brain. Oncogene.

[35] Stine Z.E., Walton Z.E., Altman B.J., Hsieh A.L., Dang C.V. (2015). MYC, metabolism, and cancer. Cancer Discov..

[36] Gnanaprakasam J.R., Wang R. (2017). MYC in regulating immunity: Metabolism and beyond. Genes.

[37] O’Neil J., Grim J., Strack P., Rao S., Tibbitts D., Winter C., Hardwick J., Welcker M., Meijerink J.P., Pieters R. (2007). FBW7 mutations in leukemic cells mediate NOTCH pathway activation and resistance to γ-secretase inhibitors. J. Exp. Med..

[38] Li Q., Pan S., Xie T., Liu H. (2021). MYC in T-cell acute lymphoblastic leukemia: Functional implications and targeted strategies. Blood Sci..

[39] Pelengaris S., Khan M., Evan G. (2002). c-MYC: More than just a matter of life and death. Nat. Rev. Cancer.

[40] Dhanasekaran R., Deutzmann A., Mahauad-Fernandez W.D., Hansen A.S., Gouw A.M., Felsher D.W. (2022). The MYC oncogene—The grand orchestrator of cancer growth and immune evasion. Nat. Rev. Clin. Oncol..

[41] Teitz T., Wei T., Valentine M.B., Vanin E.F., Grenet J., Valentine V.A., Behm F.G., Look A.T., Lahti J.M., Kidd V.J. (2000). Caspase 8 is deleted or silenced preferentially in childhood neuroblastomas with amplification of MYCN. Nat. Med..

[42] Wu K.-J., Grandori C., Amacker M., Simon-Vermot N., Polack A., Lingner J., Dalla-Favera R. (1999). Direct activation of TERT transcription by c-MYC. Nat. Genet..

[43] Long H., Hou Y., Li J., Song C., Ge Z. (2023). Azacitidine is synergistically lethal with XPO1 inhibitor selinexor in acute myeloid leukemia by targeting XPO1/eIF4E/c-MYC signaling. Int. J. Mol. Sci..

[44] Qie Z., Ma L., Tan J., Peng X. (2025). Selinexor in acute myeloid leukemia: Therapeutic applications and current challenges. Front. Pharmacol..

[45] Xiao D., Yue M., Su H., Ren P., Jiang J., Li F., Hu Y., Du H., Liu H., Qing G. (2016). Polo-like kinase-1 regulates Myc stabilization and activates a feedforward circuit promoting tumor cell survival. Mol. Cell.

[46] Man E., Evran S. (2023). Deacetylation of Histones and Non-histone Proteins in Inflammatory Diseases and Cancer Therapeutic Potential of Histone Deacetylase Inhibitors. Curr. Genom..

[47] Lernoux M., Schnekenburger M., Losson H., Vermeulen K., Hahn H., Gérard D., Lee J.-Y., Mazumder A., Ahamed M., Christov C. (2020). Novel HDAC inhibitor MAKV-8 and imatinib synergistically kill chronic myeloid leukemia cells via inhibition of BCR-ABL/MYC-signaling: Effect on imatinib resistance and stem cells. Clin. Epigenet..

[48] Donati G., Amati B. (2022). MYC and therapy resistance in cancer: Risks and opportunities. Mol. Oncol..

[49] Ju X., Zhang H., Zhou Z., Wang Q. (2020). Regulation of PD-L1 expression in cancer and clinical implications in immunotherapy. Am. J. Cancer Res..

[50] Hon K.W., Zainal Abidin S.A., Othman I., Naidu R. (2021). The crosstalk between signaling pathways and cancer metabolism in colorectal cancer. Front. Pharmacol..

[51] Li Y., Wang Y., Liu Z., Guo X., Miao Z., Ma S. (2020). Atractylenolide I induces apoptosis and suppresses glycolysis by blocking the JAK2/STAT3 signaling pathway in colorectal cancer cells. Front. Pharmacol..

[52] Godet A.-C., David F., Hantelys F., Tatin F., Lacazette E., Garmy-Susini B., Prats A.-C. (2019). IRES trans-acting factors, key actors of the stress response. Int. J. Mol. Sci..

[53] Taniguchi K., Sugito N., Kumazaki M., Shinohara H., Yamada N., Matsuhashi N., Futamura M., Ito Y., Otsuki Y., Yoshida K. (2015). Positive feedback of DDX6/c-Myc/PTB1 regulated by miR-124 contributes to maintenance of the Warburg effect in colon cancer cells. Biochim. Biophys. Acta BBA Mol. Basis Dis..

[54] Fang Y., Shen Z.-Y., Zhan Y.-Z., Feng X.-C., Chen K.-L., Li Y.-S., Deng H.-J., Pan S.-M., Wu D.-H., Ding Y. (2019). CD36 inhibits β-catenin/c-myc-mediated glycolysis through ubiquitination of GPC4 to repress colorectal tumorigenesis. Nat. Commun..

[55] Böttcher M., Bruns H., Völkl S., Lu J., Chartomatsidou E., Papakonstantinou N., Mentz K., Büttner-Herold M., Zenz T., Herling M. (2021). Control of PD-L1 expression in CLL-cells by stromal triggering of the Notch-c-Myc-EZH2 oncogenic signaling axis. J. Immunother. Cancer.

[56] Brusa D., Serra S., Coscia M., Rossi D., Gaidano G., Inghirami G., Vaisitti T., Deaglio S. (2012). The PD-1/PD-L1 axis contributes to T cell dysfunction in chronic lymphocytic leukemia. Blood.

[57] Yan J., Ng S.-B., Tay J.L.-S., Lin B., Koh T.L., Tan J., Selvarajan V., Liu S.-C., Bi C., Wang S. (2013). EZH2 overexpression in natural killer/T-cell lymphoma confers growth advantage independently of histone methyltransferase activity. Blood J. Am. Soc. Hematol..

[58] Kosalai S.T., Morsy M.H.A., Papakonstantinou N., Mansouri L., Stavroyianni N., Kanduri C., Stamatopoulos K., Rosenquist R., Kanduri M. (2019). EZH2 upregulates the PI3K/AKT pathway through IGF1R and MYC in clinically aggressive chronic lymphocytic leukaemia. Epigenetics.

[59] Gao F.-Y., Li X.-T., Xu K., Wang R.-T., Guan X.-X. (2023). c-MYC mediates the crosstalk between breast cancer cells and tumor microenvironment. Cell Commun. Signal..

[60] Atefi M., Avramis E., Lassen A., Wong D.J., Robert L., Foulad D., Cerniglia M., Titz B., Chodon T., Graeber T.G. (2014). Effects of MAPK and PI3K pathways on PD-L1 expression in melanoma. Clin. Cancer Res..

[61] Kadry M.O., Abdel Hamid A.-H.Z., Abdel-Megeed R.M. (2024). Collaboration of Hprt/K-RAS/c-Myc mutation in the oncogenesis of T-lymphocytic leukemia: A comparative study. Future Sci. OA.

[62] Jung P., Hermeking H. (2009). The c-MYC-AP4-p21 cascade. Cell Cycle.

[63] Chiang M.Y., Xu L., Shestova O., Histen G., L’Heureux S., Romany C., Childs M.E., Gimotty P.A., Aster J.C., Pear W.S. (2008). Leukemia-associated NOTCH1 alleles are weak tumor initiators but accelerate K-ras–initiated leukemia. J. Clin. Investig..

[64] Dong J., Naito M., Tsuruo T. (1997). c-Myc plays a role in cellular susceptibility to death receptor-mediated and chemotherapy-induced apoptosis in human monocytic leukemia U937 cells. Oncogene.

[65] Sedoris K.C., Thomas S.D., Miller D.M. (2010). Hypoxia induces differential translation of enolase/MBP-1. BMC Cancer.

[66] Kirtonia A., Ashrafizadeh M., Zarrabi A., Hushmandi K., Zabolian A., Bejandi A.K., Rani R., Pandey A.K., Baligar P., Kumar V. (2022). Long noncoding RNAs: A novel insight in the leukemogenesis and drug resistance in acute myeloid leukemia. J. Cell. Physiol..

[67] Shagerdi Esmaeli N., Asadi S., Bashash D., Salari S., Hamidpour M. (2023). Involvement Value of FLT-3, c-Myc, STAT3, p27, and HOTAIR Gene Expression in Acute Myeloid Leukemia Patients: A Molecular Perspective to a Novel Leukemogenesis Mechanism. Int. J. Hematol. Oncol. Stem Cell Res..

[68] Hay J., Tarafdar A., Holroyd A.K., Moka H.A., Dunn K.M., Alshayeb A., Lloyd B.H., Cassels J., Malik N., Khan A.F. (2022). PKCβ facilitates leukemogenesis in chronic lymphocytic leukaemia by promoting constitutive BCR-mediated signalling. Cancers.

[69] Amatori S., Persico G., Cantatore F., Rusin M., Formica M., Giorgi L., Macedi E., Casciaro F., Errico Provenzano A., Gambardella S. (2023). Small molecule-induced epigenomic reprogramming of APL blasts leading to antiviral-like response and c-MYC downregulation. Cancer Gene Ther..

[70] Conserva M.R., Redavid I., Anelli L., Zagaria A., Tarantini F., Cumbo C., Tota G., Parciante E., Coccaro N., Minervini C.F. (2023). IKAROS in acute leukemia: A positive influencer or a mean hater?. Int. J. Mol. Sci..

[71] Wang H., Qiu Y., Zhang H., Chang N., Hu Y., Chen J., Hu R., Liao P., Li Z., Yang Y. (2023). Histone acetylation by HBO1 (KAT7) activates Wnt/β-catenin signaling to promote leukemogenesis in B-cell acute lymphoblastic leukemia. Cell Death Dis..

[72] Bogdanov K., Kudryavtseva E., Fomicheva Y., Churkina I., Lomaia E., Girshova L., Osipov Y., Zaritskey A. (2023). Shift of N-MYC Oncogene expression in AML patients carrying the FLT3-ITD mutation. Pathophysiology.

[73] Delgado M.D., León J. (2010). Myc roles in hematopoiesis and leukemia. Genes Cancer.

[74] Xie T., Sun P., Huang H., Li Q., Liu H., Jiang J. (2025). PAK4 phosphorylates and stabilizes MYC to promote acute myeloid leukemia. Cell Insight.

[75] Militi S., Nibhani R., Pook M., Pauklin S. (2025). SMAD2/3-SMYD2 and developmental transcription factors cooperate with cell-cycle inhibitors to guide tissue formation. Protein Cell.

[76] Subramanian A., Tamayo P., Mootha V.K., Mukherjee S., Ebert B.L., Gillette M.A., Paulovich A., Pomeroy S.L., Golub T.R., Lander E.S. (2005). Gene set enrichment analysis: A knowledge-based approach for interpreting genome-wide expression profiles. Proc. Natl. Acad. Sci. USA.

[77] Song J., Liu W., Xiao X., Song J., Wang C., Gajendran B., Wei X., Yang C., Chen Y., Yang Y. (2025). Rocaglamide reprograms glucose metabolism in erythroleukemic cells via c-MYC transcriptional regulation of TXNIP and HK2. J. Ethnopharmacol..

[78] Zehtabcheh S., Sheikh-Zeineddini N., Yousefi A.-M., Bashash D. (2024). Anti-Leukemic Effects of Small Molecule Inhibitor of c-Myc (10058-F4) on Chronic Myeloid Leukemia Cells. Asian Pac. J. Cancer Prev. APJCP.

[79] Bullinger L., Valk P.J.M. (2005). Gene Expression Profiling in Acute Myeloid Leukemia. J. Clin. Oncol..

[80] Ellakwa D.E.-S., Abdelmalek M.A., Mostafa M.M., Ellakwa T.E., Wadan A.-H.S. (2025). MircoRNAs predict and modulate responses to chemotherapy in leukemic patients. Naunyn-Schmiedebergs Arch. Pharmacol..

[81] Rasheed N., Nounou H., Eltabakh S., Hamed N., Ayman Ahmed Darwish A. (2024). The Role of β-Catenin and c-Myc Dysregulation in Acute Myeloid Leukemia: A Study of Egyptian Patients. Future Perspect. Med. Pharm. Environ. Biotechnol..

[82] Li T., Lin T., Zhu J., Zhou M., Fan S., Zhou H., Mu Q., Sheng L., Ouyang G. (2023). Prognostic and therapeutic implications of iron-related cell death pathways in acute myeloid leukemia. Front. Oncol..

[83] Gasic V., Karan-Djurasevic T., Pavlovic D., Zukic B., Pavlovic S., Tosic N. (2022). Diagnostic and therapeutic implications of long non-coding RNAs in leukemia. Life.

[84] Park S., Sater A.H.A., Fahrmann J.F., Irajizad E., Cai Y., Katayama H., Vykoukal J., Kobayashi M., Dennison J.B., Garcia-Manero G. (2022). Novel UHRF1-MYC axis in acute lymphoblastic leukemia. Cancers.

[85] Rafatpanah H., Golizadeh M., Mahdifar M., Mahdavi S., Iranshahi M., Rassouli F.B. (2023). Conferone, a coumarin from Ferula flabelliloba, induced toxic effects on adult T-cell leukemia/lymphoma cells. Int. J. Immunopathol. Pharmacol..

[86] Zerehpoosh F.B., Farahmandfar M., Sharifi G., Rezaei O., Gachkar L. (2025). Immunohistochemical evaluation of CD10, BCL6, BCL2, MUM1 and MYC in diffuse large B-cell brain lymphoma; diagnostic and prognostic significance. Immunopathol. Persa.

[87] Alves R., Gonçalves A.C., Rutella S., Almeida A.M., De Las Rivas J., Trougakos I.P., Sarmento Ribeiro A.B. (2021). Resistance to tyrosine kinase inhibitors in chronic myeloid leukemia—From molecular mechanisms to clinical relevance. Cancers.

[88] Ysebaert L., Mouchel P.-L., Laurent C., Quillet-Mary A. (2025). The multi-faceted roles of MYC in the prognosis of chronic lymphocytic leukemia. Leuk. Lymphoma.

[89] Xu T., Shen Y., Guo R., Luo C., Niu Y., Luo Z., Zhu Z., Wu Z., Zhao X., Luo H. (2024). Mutual regulation between histone methyltransferase Suv39h1 and the Wnt/β-catenin signaling pathway promoted cell proliferation and inhibited apoptosis in bone marrow mesenchymal stem cells exposed to hydroquinone. Toxicology.

[90] Dabbaghipour R., Khaze Shahgoli V., Safaei S., Amini M., Tabei S., Shanehbandi D., Rahbar Farzam O., Baradaran B., Entezam M. (2024). siRNA-mediated downregulation of BATF3 diminished proliferation and induced apoptosis through downregulating c-Myc expression in chronic myelogenous leukemia cells. Mol. Biol. Rep..

[91] Fukasawa K., Kadota T., Horie T., Tokumura K., Terada R., Kitaguchi Y., Park G., Ochiai S., Iwahashi S., Okayama Y. (2021). CDK8 maintains stemness and tumorigenicity of glioma stem cells by regulating the c-MYC pathway. Oncogene.

[92] Li S., Chen G., Huang X., Zhang Y., Shen S., Feng H., Li Y. (2024). c-Myc alone is enough to reprogram fibroblasts into functional macrophages. J. Hematol. Oncol..

[93] Abd G.M., Laird M.C., Ku J.C., Li Y. (2023). Hypoxia-induced cancer cell reprogramming: A review on how cancer stem cells arise. Front. Oncol..

[94] Joshi P., Keyvani Chahi A., Liu L., Moreira S., Vujovic A., Hope K.J. (2024). RNA binding protein-directed control of leukemic stem cell evolution and function. Hemasphere.

[95] Fergany A., Hassanein K.M., Hameed M.R.A., Kamel A.M., Zahran A.M. (2025). CD34+ CD38-stem cells and CD34+ CD38+ progenitor cells as markers of chemotherapy response in acute myeloid leukemia patients. Immunopathol. Persa.

[96] Zhang X., Peng P., Bao L.-W., Zhang A.-Q., Yu B., Li T., Lei J., Zhang H.-H., Li S.-Z. (2024). Ubiquitin-Specific Protease 1 Promotes Bladder Cancer Progression by Stabilizing c-MYC. Cells.

[97] Willard P.A., Kornbluth J. (2025). The ubiquitin ligase NKLAM promotes apoptosis and suppression of cell growth. J. Biol. Chem..

[98] Sivamani Y., Venkataramanappa G., Reddy H.N., Deshpande S.N., Gopal K., Elayaperumal S., Rao D., Bhattacharya D., Das P.K., Biswas S.D. (2025). Chapter 6-Human genome and genomic variations associated with diseases. Advancing Science and Innovation in Healthcare Research.

[99] Park J.H. (2025). Functional Analysis of Fnip1 and Sox6 on Chicken Muscle Fiber Formation. Master’s Thesis.

[100] Lee J., Kim J.-H., Lee Y.J., Oh J.J., Han Y.J., Jung J.H. (2025). Elucidating the Role of CNOT2 in Regulating Cancer Cell Growth via the Modulation of p53 and c-Myc Expression. Curr. Issues Mol. Biol..

[101] Kirsner R.S., Pastar I., Krambrink A., Lev-Tov H., Burgess J.L., Kolenic G., Jozic I., Catanuto P., Marjanovic J., Jones T.L. (2025). Evaluation of c-Myc and Phosphorylated Glucocorticoid Receptor (p-GR) for Predicting Diabetic Foot Ulcer Healing—A Diabetic Foot Consortium Study. Wound Repair Regen..

[102] Han Q., Zhou Y., Dong Z., Wang W., Wang M., Pang M., Song X., Chen B., Zheng A. (2025). SNORA47 affects stemness and chemotherapy sensitivity via EBF3/RPL11/c-Myc axis in luminal A breast cancer. Mol. Med..

[103] Zhang S., Kipps T.J. (2014). The pathogenesis of chronic lymphocytic leukemia. Annu. Rev. Pathol. Mech. Dis..

[104] Liu Y., Shi M., He X., Cao Y., Liu P., Li F., Zou S., Wen C., Zhan Q., Xu Z. (2022). LncRNA-PACERR induces pro-tumour macrophages via interacting with miR-671-3p and m6A-reader IGF2BP2 in pancreatic ductal adenocarcinoma. J. Hematol. Oncol..

[105] Han Y., Li S., Oyang L., Cui S., Zhang W., Yang W., Peng M., Tan S., Xia L., Lin J. (2025). Novel insights into lncRNAs as key regulators of post-translational modifications in cancer: Mechanisms and therapeutic potential. Cell. Oncol..

[106] Gu K., May H.A., Kang M.H. (2024). Targeting molecular signaling pathways and cytokine responses to modulate c-MYC in acute myeloid leukemia. Front. Biosci. (Schol. Ed.).

[107] Yu J., Liu D., Yuan Y., Sun C., Su Z. (2025). Rethinking MYC inhibition: A multi-dimensional approach to overcome cancer’s master regulator. Front. Cell Dev. Biol..

[108] Iudin M.S., Khodarovich Y.M., Varizhuk A.M., Tsvetkov V.B., Severov V.V. (2025). A minireview on BET inhibitors: Beyond bromodomain targeting. Biomedicines.

[109] Davids M.S., Brander D.M., Alvarado-Valero Y., Diefenbach C.S., Egan D.N., Dinner S.N., Javidi-Sharifi N., Al Malki M.M., Begna K.H., Bhatt V.R. (2025). A phase 1 study of the CDK9 inhibitor voruciclib in relapsed/refractory acute myeloid leukemia and B-cell malignancies. Blood Adv..

[110] Stipp M.C., Acco A. (2025). c-Myc-targeted therapy in breast cancer: A review of fundamentals and pharmacological Insights. Gene.

[111] Whitfield J.R., Soucek L. (2025). MYC in cancer: From undruggable target to clinical trials. Nat. Rev. Drug Discov..

[112] Madden S.K., de Araujo A.D., Gerhardt M., Fairlie D.P., Mason J.M. (2021). Taking the Myc out of cancer: Toward therapeutic strategies to directly inhibit c-Myc. Mol. Cancer.

[113] Wang C., Zhang J., Yin J., Gan Y., Xu S., Gu Y., Huang W. (2021). Alternative approaches to target Myc for cancer treatment. Signal Transduct. Target. Ther..

[114] Thumpati P., Rai S.N., Prajapati C., Ramakrishna K., Singh S.K. (2025). Targeting c-MYC G-quadruplexes for cancer treatment with small molecules. Sci. Pharm..

[115] Dhimitriu R., Tsimpili H., Zoidis G. (2025). Key breakthroughs in small molecule MYC inhibitors. Future Med. Chem..

[116] Zhang Y., Ye M., Luan X., Sun Z., Zhang W.D. (2025). Exploiting replication stress for synthetic lethality in MYC-driven cancers. Am. J. Cancer Res..

[117] Popov D. (2024). The Intersection of Viral Infections, RNA Alterations, and Cancer: Mechanisms and Treatment Strategies. Viral Carcinogenesis and RNA: Biomarkers, Mechanisms, and RNA-Based Therapies.

[118] Huang S., Li Z., Lin W., Xie R., Huang H. (2025). RNA Epigenetics in Cancer: Current Knowledge and Therapeutic Implications. MedComm.

[119] Langdon S.P., Kay C., Um I.H., Dodds M., Muir M., Sellar G., Kan J., Gourley C., Harrison D.J. (2019). Evaluation of the dual mTOR/PI3K inhibitors Gedatolisib (PF-05212384) and PF-04691502 against ovarian cancer xenograft models. Sci. Rep..

[120] Kasner M., Luger S.M., Jeschke G.R., Mick R., Carroll M., Perl A.E. (2011). Single-Cell Pharmacodynamic Monitoring of S6 Ribosomal Protein in AML Blasts During Trials Combining Sirolimus and Intensive Chemotherapy: Target Inhibition Enhances Response. Blood.

[121] Vick E.J., Hassan A., Choi K., Bennett J., Muto T., Clough C.A., Culver-Cochran A.E., Hueneman K., Bolanos L.C., Wunderlich M. (2025). Targeting of IRAK4 and GSPT1 enhances therapeutic efficacy in AML via c-Myc destabilization: ACUTE MYELOID LEUKEMIA. Leukemia.

[122] Jędraszek K., Malczewska M., Parysek-Wojcik K., Lejman M. (2022). Resistance mechanisms in pediatric B-cell acute lymphoblastic leukemia. Int. J. Mol. Sci..

[123] Mojtahedi H., Yazdanpanah N., Rezaei N. (2021). Chronic myeloid leukemia stem cells: Targeting therapeutic implications. Stem Cell Res. Ther..

[124] Bai L., Zhou L., Han W., Chen J., Gu X., Hu Z., Yang Y., Li W., Zhang X., Niu C. (2023). BAX as the mediator of C-MYC sensitizes acute lymphoblastic leukemia to TLR9 agonists. J. Transl. Med..

[125] Stelmach P., Trumpp A. (2023). Leukemic stem cells and therapy resistance in acute myeloid leukemia. Haematologica.

[126] Wang P.-H., Hu C.-H., Fan J.-Q., He J.-J., Deng T.-F., Xu Y.-L., Dai Y.-J., Wang S.-Q. (2025). Innovative evaluation of selinexor and JQ1 synergy in leukemia therapy via C-MYC inhibition. J. Transl. Med..

[127] Li Q., Wang F., Xiang X., Zhao L., Li X., Qu Y., Huang J., Yang Y., Dai Y., Shuai X. (2025). Chidamide and cytarabine synergistically treat acute myeloid leukemia: Inhibiting ribosome biogenesis via the MYC-RRP9 pathway. Cell Death Dis..

[128] Arroyo-Berdugo Y., Di Mambro A., Behrends V., Sahai M.A., Cozzuto L., Zollo I., Ponomarenko J., Williams O., Gribben J., Calle Y. (2025). Exploiting PP2A dependent and independent effects of forskolin for therapeutic targeting of KMT2A (MLL)-rearranged acute leukaemia. Br. J. Pharmacol..

[129] Bunjes D. (2025). Radioimmunotherapy of acute myeloid leukemia: A critical assessment of its prospects and limitations. Expert Rev. Hematol..

[130] Haque M., Shakil M.S., Mahmud K.M. (2023). The promise of nanoparticles-based radiotherapy in cancer treatment. Cancers.

[131] Webster M., Podgorsak A., Li F., Zhou Y., Jung H., Yoon J., Dona Lemus O., Zheng D. (2025). New approaches in radiotherapy. Cancers.

[132] Paganetti H. (2023). A review on lymphocyte radiosensitivity and its impact on radiotherapy. Front. Oncol..

[133] Rafat A., Asl K.D., Mazloumi Z., Movassaghpour A.A., Talebi M., Shanehbandi D., Farahzadi R., Nejati B., Charoudeh H.N. (2022). Telomerase inhibition on acute myeloid leukemia stem cell induced apoptosis with both intrinsic and extrinsic pathways. Life Sci..

[134] Bai X., Zhou Y., Yokota Y., Matsumoto Y., Zhai B., Maarouf N., Hayashi H., Carlson R., Zhang S., Sousa A. (2022). Adaptive antitumor immune response stimulated by bio-nanoparticle based vaccine and checkpoint blockade. J. Exp. Clin. Cancer Res..

[135] Li J., Dong T., Wu Z., Zhu D., Gu H. (2023). The effects of MYC on tumor immunity and immunotherapy. Cell Death Discov..

[136] Casey S.C., Baylot V., Felsher D.W. (2018). The MYC oncogene is a global regulator of the immune response. Blood J. Am. Soc. Hematol..

[137] Khaldoyanidi S., Nagorsen D., Stein A., Ossenkoppele G., Subklewe M. (2021). Immune biology of acute myeloid leukemia: Implications for immunotherapy. J. Clin. Oncol..

[138] Bahramloo M., Shahabi S.A., Kalarestaghi H., Rafat A., Mazloumi Z., Samimifar A., Asl K.D. (2024). CAR-NK cell therapy in AML: Current treatment, challenges, and advantage. Biomed. Pharmacother..

[139] Upchurch M.D., Muluneh B. (2024). Treatment adherence and adverse event management in chronic lymphocytic leukemia: Challenges and strategies for the future. Expert Rev. Clin. Pharmacol..

[140] Gómez-De León A., Demichelis-Gómez R., da Costa-Neto A., Gómez-Almaguer D., Rego E.M. (2023). Acute myeloid leukemia: Challenges for diagnosis and treatment in Latin America. Hematology.

[141] Obeagu E.I. (2025). Advancing Leukemia Diagnosis and Treatment: WHO-Supported Laboratory Innovations in Africa-A Narrative Review. Blood Lymphat. Cancer Targets Ther..

[142] Salvati A., Melone V., Giordano A., Lamberti J., Palumbo D., Palo L., Rea D., Memoli D., Simonis V., Alexandrova E. (2025). Multi-omics based and AI-driven drug repositioning for epigenetic therapy in female malignancies. J. Transl. Med..

[143] Chen B., Gao Z., Chen L., Zhang H., Lin Y., Li J. (2025). Detection of leukemia gene fusions on DNA-level through targeted Next-Generation Sequencing. PLoS ONE.

[144] Hallek M. (2019). Chronic lymphocytic leukemia: 2020 update on diagnosis, risk stratification and treatment. Am. J. Hematol..

[145] Sánchez Suárez M.D.M., Martín Roldán A., Alarcón-Payer C., Rodríguez-Gil M.Á., Poquet-Jornet J.E., Puerta Puerta J.M., Jiménez Morales A. (2024). Treatment of Chronic Lymphocytic Leukemia in the Personalized Medicine Era. Pharmaceutics.

[146] Islam S.U., Ahmed M.B., Ahsan H., Lee Y.S., Shehzad A. (2022). Role of Biomarkers in Personalized Medicine. Cancer Biomarkers in Diagnosis and Therapeutics.

[147] Sergio I., Varricchio C., Squillante F., Cantale Aeo N.M., Campese A.F., Felli M.P. (2024). Notch Inhibitors and BH3 Mimetics in T-Cell Acute Lymphoblastic Leukemia. Int. J. Mol. Sci..

[148] Shi Y., Wang X., Meng Y., Ma J., Zhang Q., Shao G., Wang L., Cheng X., Hong X., Wang Y. (2021). A novel mechanism of endoplasmic reticulum stress-and c-Myc-degradation-mediated therapeutic benefits of antineurokinin-1 receptor drugs in colorectal cancer. Adv. Sci..

[149] Zhang K., Gao L., Wang J., Chu X., Zhang Z., Zhang Y., Fang F., Tao Y., Li X., Tian Y. (2022). A novel BRD family PROTAC inhibitor dBET1 exerts great anti-cancer effects by targeting c-MYC in acute myeloid leukemia cells. Pathol. Oncol. Res..

[150] Rahgoshay M., Atashi A., Vaezi M., Ajorloo M., Amini-Kafiabad S., Ahmadvand M. (2025). Engineered exosomes: Advanced nanocarriers for targeted therapy and drug delivery in hematological malignancies. Cancer Nanotechnol..

[151] Weber L.I., Hartl M. (2023). Strategies to target the cancer driver MYC in tumor cells. Front. Oncol..

[152] Rafat A., Dizaji Asl K., Mazloumi Z., Movassaghpour A.A., Farahzadi R., Nejati B., Nozad Charoudeh H. (2022). Telomerase-based therapies in haematological malignancies. Cell Biochem. Funct..

[153] Hou Y., Dovat K., Dovat S., Song C., Ge Z. (2023). Pb1784: Cladribine Synergizes the Effect of Venetoclax on Cell Proliferation Arrest and Apoptosis by Targeting DNA-Pkcs/C-Myc Signaling in Acute Myeloid Leukemia. Hemasphere.

